# Algorithm for predicting valvular heart disease from heart sounds in an unselected cohort

**DOI:** 10.3389/fcvm.2023.1170804

**Published:** 2024-01-24

**Authors:** Per Niklas Waaler, Hasse Melbye, Henrik Schirmer, Markus Kreutzer Johnsen, Tom Donnem, Johan Ravn, Stian Andersen, Anne Herefoss Davidsen, Juan Carlos Aviles Solis, Michael Stylidis, Lars Ailo Bongo

**Affiliations:** ^1^ Department of Computer Science, UiT The Arctic University of Norway, Tromsø, Norway; ^2^ General Practice Research Unit, Department of Community Medicine, UiT The Arctic University of Norway, Tromsø, Norway; ^3^ Department of Cardiology, Akershus University Hospital, Oslo, Norway; ^4^ Institute of Clinical Medicine, Cardiovascular Research Group, University of Oslo, Oslo, Norway; ^5^ Department of Clinical Medicine, UiT The Arctic University of Norway, Tromsø, Norway; ^6^ Medsensio AS, Oslo, Norway; ^7^ Department of Oncology, University Hospital of North Norway, Tromsø, Norway; ^8^ Sørbyen Legesenter, Tromsø, Norway

**Keywords:** general population, screening, heart sounds, machine learning, murmur, aortic stenosis, valvular heart disease

## Abstract

**Objective:**

This study aims to assess the ability of state-of-the-art machine learning algorithms to detect valvular heart disease (VHD) from digital heart sound recordings in a general population that includes asymptomatic cases and intermediate stages of disease progression.

**Methods:**

We trained a recurrent neural network to predict murmurs from heart sound audio using annotated recordings collected with digital stethoscopes from four auscultation positions in 2,124 participants from the Tromsø7 study. The predicted murmurs were used to predict VHD as determined by echocardiography.

**Results:**

The presence of aortic stenosis (AS) was detected with a sensitivity of 90.9%, a specificity of 94.5%, and an area under the curve (AUC) of 0.979 (CI: 0.963–0.995). At least moderate AS was detected with an AUC of 0.993 (CI: 0.989–0.997). Moderate or greater aortic and mitral regurgitation (AR and MR) were predicted with AUC values of 0.634 (CI: 0.565–703) and 0.549 (CI: 0.506–0.593), respectively, which increased to 0.766 and 0.677 when clinical variables were added as predictors. The AUC for predicting symptomatic cases was higher for AR and MR, 0.756 and 0.711, respectively. Screening jointly for symptomatic regurgitation or presence of stenosis resulted in an AUC of 0.86, with 97.7% of AS cases (*n* = 44) and all 12 MS cases detected.

**Conclusions:**

The algorithm demonstrated excellent performance in detecting AS in a general cohort, surpassing observations from similar studies on selected cohorts. The detection of AR and MR based on HS audio was poor, but accuracy was considerably higher for symptomatic cases, and the inclusion of clinical variables improved the performance of the model significantly.

## Introduction

Valvular heart disease (VHD) is a major source of dysfunction, reduced quality of life, early death, and increased healthcare costs (1–3). The prevalence of VHD in the United States is estimated to be 2.2% in the general population and 13.3% among those aged 75 years or older (4). The prevalence of aortic stenosis (AS), the most common form of VHD in developed countries, which has a poor prognosis when left untreated, was estimated to 12.4% in those 75 years or older based on data from European countries and North America in 2013 (5). Prevalence rates of mitral regurgitation (MR) and aortic regurgitation (AR) are also strongly age-dependent (6, 7), and with the projected growth of the elderly population and the rapid increase of VHD prevalence with age, the societal burdens of VHD are expected to increase considerably (2).

Although studies have indicated that a stethoscope can be a cost-efficient screening tool for cardiovascular disease, its potential utility is limited by the increasing time constraints imposed on doctors, the declining skill of healthcare providers in performing cardiac auscultation (8–10), and low interrater agreement (11). While the echocardiogram is the gold standard for detecting VHD, it is expensive (12) and requires highly trained personnel to analyze its results and therefore cannot replace the stethoscope as a front-line screening tool. Effective treatment of VHD exists (13), which further highlights the need for inexpensive and scalable screening methods. Deep learning models have shown promising results in HS classification over recent years (14, 15), and successful implementation of such automated methods could offer several advantages over manually performed auscultation. Machine learning algorithms have the potential to improve upon auscultation performance in clinical settings as they are not subject to many of the potential sources of random error that influence human judgment, such as stress or poor sleep. Algorithms can also minimize the influence of random errors in training by extracting trends rather than reproducing each label (i.e., overfitting); thus, they can not only match but also potentially exceed the performance of the annotators that generate the data, especially when multiple raters are used since data aggregation can produce more reliable ratings. Also, unlike humans, time available for practice is a non-issue. Finally, algorithms can grant widespread access to the performance of the most skilled practitioners in ideal settings where they perform at their best.

Although impressive ML results in cohorts of patients with known heart disease and healthy controls have been achieved, the ability of state-of-the-art methods to effectively screen for VHD using heart sounds in a general population has not been demonstrated. Due to the rarity of significant VHD relative to the large amounts of data required to train deep neural networks, the standard practice in automated HS analysis research is to enroll study participants from hospitals. It is plausible that this practice results in a high prevalence of more detectable and/or symptomatic cases, which in turn might bias test metrics (16). This is a potential problem since the degree of detectability and the presence of symptoms are not always reliable indicators of VHD severity (17–19). As such, it is important to assess the impact of the procedure for enrolling study participants on algorithm performance metrics. Our primary aim was to explore this effect and investigate the potential of machine learning technology as a front-line screening tool for detecting undiagnosed VHD from heart sounds in a general population. To assess this potential, we developed a murmur detection algorithm using state-of-the-art methods, which we then used to screen for left-sided VHD. We aimed to establish which pathological cases are reliably detected and how detectability relates to disease severity and presence of symptoms. Since our dataset uniquely had four murmur-annotated heart sound recordings per person, we also investigated how to efficiently use murmur grade predictions to predict AS through a multivariate regression analysis where we take predicted murmur grades as input variables**.** Finally, we aimed to establish whether combining clinical variables with heart sound-based algorithm predictions can improve VHD screening using a multivariate regression analysis.

## Methods

### Study design

**Figure 1:**
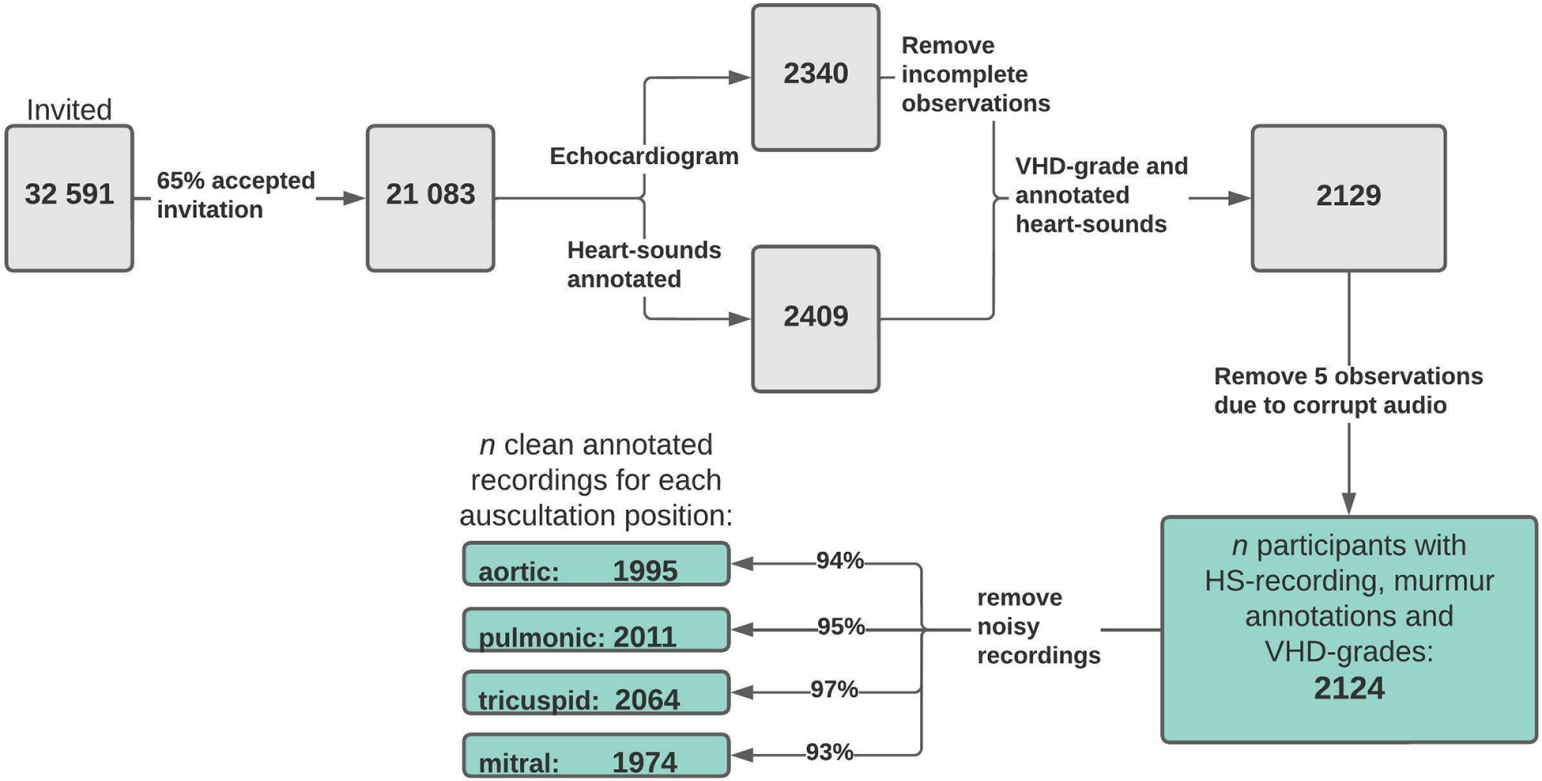
Data flowchart. Flowchart overview of how the final study dataset was formed and how many samples were excluded due to noisy or incomplete data. The values at the end show the number of usable (absence of considerable noise) recordings annotated with murmur grades and grades of AR, MR, AS, and MS for each auscultation position. VHD, valvular heart disease; AR, aortic regurgitation; MR, mitral regurgitation; AS, aortic stenosis; MS, mitral stenosis.

The Tromsø study is an ongoing population-based prospective study that was initiated in 1974 in the municipality of Tromsø, Norway. The seventh and most recent survey was carried out in 2015 and 2016, which provided the data used in this study (20). All inhabitants of Tromsø aged 40 years or older (age ranging from 40 to 99 years) were invited to participate in the study. The Tromsø study is the largest study of its kind in Norway, with 32,591 people invited to participate in its seventh iteration, of which 64.7% participated. A randomly selected subset of the participants underwent echocardiography examinations (*n* = 2,340) and physical examinations, which included cardiac auscultation (*n* = 2,409) (21). In total, 2,132 participants underwent both echocardiography and heart sound recording, and these make up the dataset examined in this study. See Figure 1 for a flowchart overview of the data collection.

All study participants received questionnaires by email, and from the questionnaires, we collected information on the following variables: breathlessness [breathlessness while (1) resting, (2) walking calmly on level ground, and (3) walking up a hill], mMRC scale (modified medical research council dyspnea scale from 0 to 4), blood pressure, chest pain, angina pectoris, smoking status, and diabetes. Questions on clinical conditions were of the form “Do you or have you had ….” For all participants, we had access to their gender, age, and body mass index (BMI), and from physical examinations, we had access to their heart rate (prior to a spirometry test) and pulse oximetry results. Percentages of data missing for these variables were 4.24% (breathlessness while resting or walking calmly on level ground), 6.03% (mMRC scale), 2.45% (high blood pressure), 1.7% (chest pain), 4.0% (angina), 1.4% (smoking status), 2.8% (diabetes), 6.12% (heart rate), and 5.93% (pulse oximetry reading). More details on the tabular data are provided in Supplementary Table S1. In the multivariate analysis (see pages 19 and 20), missing questionnaire answers were filled in using the most frequent answer. Given the low prevalence of missing values, we did not believe this to be a significant source of bias.

### Heart sound recording

In the Tromsø7 study, a microphone was attached (Sennheiser MKE 2-EW) inside the tube of a stethoscope (Littmann Classic II), positioned 10 cm from the chestpiece. The microphone was connected to a wireless system (Sennheiser EW 112-P G3-G) that transmitted the sound signal to a computer via an external sound card (Scarlett 2i2, Focusrite Audio). Heart sounds were recorded for 10 s. Audio files were recorded in the “.wav” format in a single monophonic channel with a depth of 16 bits at a rate of 44,100 Hz. Participants were sitting in a chair and were asked to breathe normally. For each participant, recordings were collected in four locations: aortic (second intercostal space, right parasternal line), pulmonic (second intercostal space, left parasternal line), tricuspid (fourth intercostal space, left parasternal line), and mitral positions (fifth intercostal space, left midclavicular line), which in the following will be referred to as positions 1–4 when convenient. More details on the recording procedure can be found in the paper by Andersen et al. (11).

### Heart sound annotation and training target

The few cases of VHD in the study cohort meant that we did not have enough statistical power in our data to both effectively develop models and estimate performance, and we therefore decided to train the deep learning model to predict murmur grades and subsequently predict VHD based on prediction of this proxy target. A comparison between models trained on VHD and murmur grades is given in the Supplementary Materials under “Training with VHD as label”. The heart sound recordings were annotated by two general practitioners (GPs) (AD and SA) working on PhD projects on heart sounds. AD is a GP specialist with 2 years of training in cardiology. Ahead of the annotation, the two GPs, a professor of cardiology (HS) and a professor of general practice (HM), independently annotated 400 recordings, discussed all disagreements, and reached a consensus on the presence and quality of murmurs. This training was reinforced at intervals throughout the annotation process when the two annotators discussed disagreements and pursued consensus on the presence of murmurs. The consensus outcomes, to which HM and HS also contributed, are not dealt with in this paper. The annotators watched spectrogram visualizations of the recordings (using Adobe Audition CS6) while classifying the heart sounds. They were blind to the echocardiography results and other information about study participants during the HS annotation.

Each recording was annotated as either normal, systolic murmur, diastolic murmur, or noise (unable to tell whether there is a murmur due to interference or low quality). Any perceived murmurs were graded on a scale from 1 (faint) to 6 (distinct), referencing the Levine scale, which is commonly used in clinical practice (22). However, the scale is not directly transferable to recordings, as grades 4–6 are associated with a palpable thrill. Therefore, in our annotated set, grades higher than 3 only reflect increases in murmur loudness and no other aspects associated with the Levine scale. A total of 2,129 participants were annotated with both murmur and VHD grades. Of these, five were removed due to corrupted audio files, resulting in an effective sample of 2,124 participants and 8,496 annotated audio files (1,416 min of audio). We trained the algorithm only on the recordings that both annotators had agreed were not noisy (recordings will hereafter simply be referred to as *noisy* and *non-noisy*). In total, there were 129 (6.1%), 113 (5.3%), 60 (2.8%), and 150 (7.1%) noisy recordings in positions 1–4, respectively. A total of 1.4% of participants had noise in all four recordings, which were excluded from the analysis. For single-recording-based prediction, only non-noisy recordings were used. For predictions using several recordings, which could have a mixture of noisy and non-noisy recordings (see the section on AS prediction using multiple recordings), noisy recordings were assigned grade 0 (murmur absent).

We used the average of the two annotators gradings to represent the murmur grade of each position. In the following, this variable is referred to simply as *the* murmur grade. By this convention, the dataset contained 465, 280, 303, and 196 cases of murmur grade > 0 in positions 1–4, respectively, and 1,244 audio recordings for which at least one annotator perceived a murmur.

### Echocardiography

The primary endpoint of this study was the presence of clinically relevant VHD determined via echocardiography. All echocardiography examinations were performed according to the American Society of Echocardiography's Guidelines using a GE Vivid E9 (GE Medical, Horten, Norway) ultrasound scanner (23). The examination was performed by an experienced echo technician before the heart sounds were annotated, and a reading of the results was performed by an experienced physician (MS) (21). AS and MS severity was graded on a scale of 0 to 3 (absent, mild, moderate, and severe), and AR and MR severity on a scale of 0 to 4 (absent, trace, mild, moderate, and severe). AS was graded by the aortic valve mean pressure gradient (AVPGmean) using cutoff values of 15 mm Hg (mild), 20 mm Hg (moderate), and 40 mm Hg (severe) (24).

### Algorithm development

The murmur detection algorithm was trained using all HS recordings; we did not distinguish between their positions during training (the model did not “know” which position a recording came from). Network training and prediction were performed in MATLAB using the MATLAB deep learning toolbox. The model and data processing used in this study were based on a study by Latif et al., in which state-of-the-art results were achieved for detecting abnormal heart sounds on the PhysioNet 2016 challenge dataset using recursive neural networks (RNNs) (15). For network architecture, we used a two-layer (50 neurons each) long-short-term-memory (LSTM) network, followed by a fully connected layer consisting of 30 neurons (Supplementary Figure S1 shows the model architecture), which finally connects to a regression layer that predicts murmur grades. See the Supplementary Materials for a performance comparison between models trained using binary and continuous labels. The initial learning rate was set to 0.002 and was halved every five epochs. To balance the ratio of murmur to non-murmur samples, we resampled from the class with murmur grade ≥ 1 until the ratio of murmur grade ≥ 1 to murmur grade < 1 was approximately 1:1.

After performing spike removal [following the steps outlined in a 2009 paper by Schmidt et al. (25)] and downsampling the signal to a sampling rate of 2,205 Hz, each recording was segmented into six overlapping (50%) blocks, each consisting of four cardiac cycles (each cycle starting and ending at the start of the first heart sound, S1). Detection of cardiac cycle boundaries was achieved using a version of the segmentation algorithm of Springer et al. (26, 27) that we modified to utilize information from multiple recordings to obtain a more robust heart-rate estimate (detailed descriptions of the modifications are given in the Supplementary Materials). For each segment, the 13 first Mel frequency cepstral coefficients (MFCCs) were computed, where we used the Hanning window function with a step size of 25 ms and a window overlap of 10 ms for computation of the spectrogram. The input units to the LSTM network were finally obtained by resizing the MFCC matrix to 13 × 200 dimensions using cubic interpolation, followed by subtracting the mean and dividing by the standard deviation of the matrix. The median predicted murmur grade over the six blocks was taken as the prediction for the whole audio recording. Supplementary Figure S2 provides a schematic overview of the steps that convert raw audio input to algorithm prediction.

### Predicting AS using multiple auscultation positions

We hypothesized that more accurate prediction could be achieved by aggregating murmur grade predictions from all four recordings in a linear multivariate predictive model rather than predicting AS using audio from a single predetermined position. In the following, these models are referred to as the *multiposition model* and *single-position model,* respectively, and we refer to the prediction using the aortic position for input as the first single-position model, and so on. As there were only 45 cases of AS ≥ 1 in the subset used for cross-validation (CV), using AS classification accuracy for model selection would likely result in overfitting. Therefore, we instead modeled AVPGmean as a function of murmur grade predictions using linear regression and based AS prediction on this model. As model candidates, we considered linear regression models that contain up to second-degree terms and noise indicator terms (taking values 0 and 1) that effectively adjust the weights of the non-noisy positions when one or more positions have noisy recordings. Model selection was performed by starting with a base model with one term for each murmur grade prediction, and then terms with low *p*-values (*p* > 0.05) were stepwise added or eliminated to find the submodel with the lowest Bayesian information criterion (BIC) value—a measure of goodness of fit that penalizes high model complexity. After the best model had been estimated by this procedure, we added, in a similar manner, the noise indicator terms. After the model had been selected, the parameters were re-estimated on the training set of each cross-validation split.

To test whether using all four recordings improved the prediction of AVPGmean, we computed the AUC for the prediction of AVPGmean > *u* across a range of thresholds (*u *= 7–30 mm Hg) using the multiple-position model and compared it to each of the single-position models. For comparison against the *i*th single-position model (the model that utilizes audio from position *i* to make predictions), we excluded observations with noise in the *i*th position.

### Performance estimation and model comparison

Due to the small number of clinically relevant cases of VHD in the dataset, we opted to rely primarily on eightfold CV to estimate performance for all predictive models. Using the same dataset for both model finetuning and performance estimation can result in overestimating model performance. Prior to performing any data analysis, we therefore set aside a holdout set containing data from 212 (∼10% of the data) participants, which contains a total of 91 cases (at the audio level) of murmur grade ≥ 1 and 55 cases murmur grade ≥ 2. We referred to the remaining set of 1,912 participants as the development set that we used for CV and model development. The tuning of the model and data-processing hyperparameters were made on the basis of murmur prediction performance and not the prediction of VHD. Finally, most decisions concerning model architecture and data processing were based on a previous study, thus minimizing overfitting as a result of fine-tuning the model to the development set. After the models were developed and results obtained, we retrained the murmur detection algorithm using the whole development set and tested for potential overfitting on the holdout set. All data splits were based on participant identification numbers to ensure independence.

### Combining clinical variables and heart sound predictions

We wanted to see whether deep learning features derived from heart sounds and traditional clinical variables combined could produce more accurate VHD predictions than either type of information in isolation. To this end, we created, for each VHD, a multivariate logistic regression model that takes as input a set of clinical variables as well as the VHD prediction by RNN models to form a predictor that incorporates both sources of input. The clinical variables we considered were age, gender, breathlessness during various activities, smoking, pulse spirometry readings, and heart rate. We also tested whether the increases in risk associated with the increase in murmur grade varies between men and women by including an interaction term. The predicted murmur grades were aggregated and entered the models as follows: the AS model used the output of the multiposition model described above, the MR model used the grade predicted from the mitral position, and the MR and MS models used the highest murmur grade for each set of four recordings (the *maximum* murmur grade). The RNN predictions were collected from the validation sets during cross-validations.

### Influence of symptoms on detectability

To test the hypothesis that symptomatic cases of AR and MR are more detectable in heart sounds than asymptomatic cases, we considered the prevalence of symptomatic cases in two subsets of AR and MR cases: those that the algorithm correctly identified and those that it missed (true positives and false negatives). If symptomatic cases tend to be more easily detected in heart sounds, we expect to see a higher prevalence of symptomatic cases in the correctly identified cases than in the missed cases. Symptomatic cases were defined as perceived breathlessness while resting or walking calmly on level ground or mMRC ≥ 2. Inferring this variable was possible for all but one participant.

### Joint screening for significant cases

Since a positive screening test must be confirmed with an echocardiography examination, an important performance metric is the ability to detect the presence of any significant VHD. To evaluate the overall ability of the algorithm to direct clinical care, we screened jointly for clinically significant cases using predicted murmur grades. For simplicity, we aggregated the predicted murmur grades by taking the maximum across the four positions and used this value as the predictor of significant cases. We defined “significant VHD” in three different ways to assess the sensitivity of performance metrics to how this class is defined: (1) grade ≥ 3 regurgitation or grade ≥ 1 stenosis, (2) grade ≥ 4 regurgitation or grade ≥ 1 stenosis, and (3) symptomatic grade ≥ 3 regurgitation or grade ≥ 1 stenosis.

### Statistical analysis

Algorithm performance was measured primarily using AUC, sensitivity, and specificity. AUC is the preferred comparison metric in this study, as it provides a summary of performance that takes into account the rate of both true positives and true negatives and is advantageous in underpowered studies where there are few positive cases since it does not require estimation of decision thresholds. Unless otherwise stated, murmur detection metrics represent average performance across auscultation positions, computed using data from all positions combined in a single dataset with pairs of recording and murmur grade. All confidence intervals (CI) and *p*-values reported in this paper correspond to a significance level of 5%, and all statistical tests are two-sided. The level of statistical significance is signified by (*), (**), and (***), which corresponds to *p*-values within the intervals (0.01, 0.05), (0.001, 0.01), and (0, 0.001), respectively.

We assumed that metrics computed on each CV validation set are independent and identically distributed. CIs for sensitivities and specificities were computed using exact methods based on the corresponding binomial distributions. Decision thresholds were automatically set (for each target separately) as the thresholds that maximized sensitivity + specificity on the CV training set under the condition that sensitivity should exceed 50%. As measures of interrater agreement, we considered ratings across all recordings jointly without distinguishing between positions and used percent agreement and Cohen's kappa to quantify agreement on murmur grade ≥ 1. We also calculated Cohen's kappa for each position separately. To contextualize the AUC for murmur detection achieved by the algorithm, we used the annotations of SA as a predictor of which recordings AD had rated as murmur grade ≥ 2 and calculated the AUC for this prediction.

## Results

After removing participants with corrupt audio files, the dataset we used in our analysis consisted of 2,124 participants who had received echocardiogram examinations and had annotated heart sounds. Table 1 shows study demographics for the study cohort. A total of 150 (7.06%) participants had AR ≥ 3 (20%), 292 (13.7%) had MR ≥ 3, 51 (2.4%) had AS ≥ 1, and 13 (0.612%) had MS ≥ 1. A total of 408 (19.2%) participants had at least one significant VHD and 44 (10.8%) of these reported symptoms. Supplementary Table S1 in Supplementary Materials provides more details on VHD prevalence and demographics.

**Table 1:** Cohort characteristics stratified by VHD.

VHD subgroup	Participants in subgroup (% of cohort)	Chest pain or angina (%)	Diabetes (%)	High blood pressure (%)	VHD symptoms (breathless) (%)	BMI (mean) (95% CI)	Heart rate (mean) (95% CI)	Age (mean) (95% CI)	Female (%)
No significant VHD	1,721 (81.0%)	141 (8.2%)	118 (7.0%)	601 (35.7%)	111 (6.4%)	27.3 (27.1–27.6)	64.5 (64.0–65.0)	62.6 (62.1–63.2)	954 (55.4%)
At least one VHD	403 (19.0%)	54 (13.5%)	21 (5.4%)	166 (42.6%)	43 (10.7%)	26.5 (26.1–26.9)	61.2 (60.2–62.2)	69.6 (68.7–70.6)	192 (47.6%)
AR ≥ 3	150 (7.1%)	20 (13.3%)	8 (5.6%)	72 (49.0%)	20 (13.4%)	26.4 (25.7–27.1)	61.0 (59.4–62.5)	71.3 (69.8–72.8)	66 (44.0%)
MR ≥ 3	292 (13.7%)	39 (13.4%)	13 (4.6%)	114 (40.7%)	33 (11.3%)	26.2 (25.8–26.7)	61.0 (59.8–62.3)	69.0 (67.8–70.1)	145 (49.7%)
AS ≥ 1	45 (2.1%)	7 (15.6%)	6 (14.0%)	25 (56.8%)	10 (22.2%)	26.8 (25.5–28.2)	62.7 (59.3–66.0)	74.4 (72.0–76.7)	16 (35.6%)
MS ≥ 1	13 (0.6%)	2 (16.7%)	2 (18.2%)	8 (66.7%)	3 (23.1%)	28.0 (25.1–31.0)	71.8 (64.9–78.6)	77.7 (74.7–80.7)	10 (76.9%)

The first column shows the number of participants in the Tromsø7 cohort within each VHD subgroup; percentages are with respect to the whole cohort. VHDs are defined by comparing the severity grade against the threshold values indicated in the corresponding row names. The other columns show the number of participants having the condition or meeting the specified criteria within each VHD subgroup, and percentages are with respect to the VHD subgroup. Symptoms of VHD are defined as shortness of breath lying down or walking calmly on level ground.

VHD, valvular heart disease; AR, aortic regurgitation; MR, mitral regurgitation; AS, aortic stenosis; MS, mitral stenosis.

### Murmur prevalence and annotator agreement

The prevalence of participants with murmur grade ≥ 1 in at least one position was 21.4% according to SA annotations and 20.1% according to AD annotations. Increasing the murmur grade threshold to ≥2, the prevalence decreased to 13.1% and 13.8%, respectively. Murmurs were most prevalent in the aortic position, with 18.6% (CI: 16.9%–20.2%) of recordings being graded as ≥1 (SA) in the aortic position compared to 11.0% (CI: 9.70%–12.4%) in the pulmonic position (Supplementary Figure S3 provides a more detailed overview of murmur annotation results for both annotators). Using SA annotations to predict recordings AD had classified as grade ≥ 2, an AUC of 0.933 was obtained. For agreement on murmur grade ≥ 1 across all recordings, Cohen's kappa was 0.717 and percent agreement was 94.2%. For specific positions, the highest Cohen's kappa was 0.733, observed in the aortic position, and the lowest was 0.666, observed in the mitral position. When defining the presence of murmur as grade ≥ 2, we found that 45.8% of the murmurs detected were *innocent* in the sense that they did not correspond to grade ≥ 1 stenosis or grade ≥ 3 regurgitation. The proportion of innocent murmurs decreased to 26.9% when grade = 2 cases of regurgitation were considered relevant.

Most murmurs were systolic, as only nine participants had any diastolic murmurs observed. There were 51 cases of at least mild AS; the annotators observed a murmur in at least one location in 47 (92.2%) of these cases, and 18 (35%) had significant MR (which can also produce a systolic murmur). Of the 47 AS cases with a murmur, 17 (36%) also had MR. See Supplementary Table S2 for more summary statistics on the relationship between the VHD grade and the presence of murmur.

### Murmur detection algorithm performance

**Figure 2:**
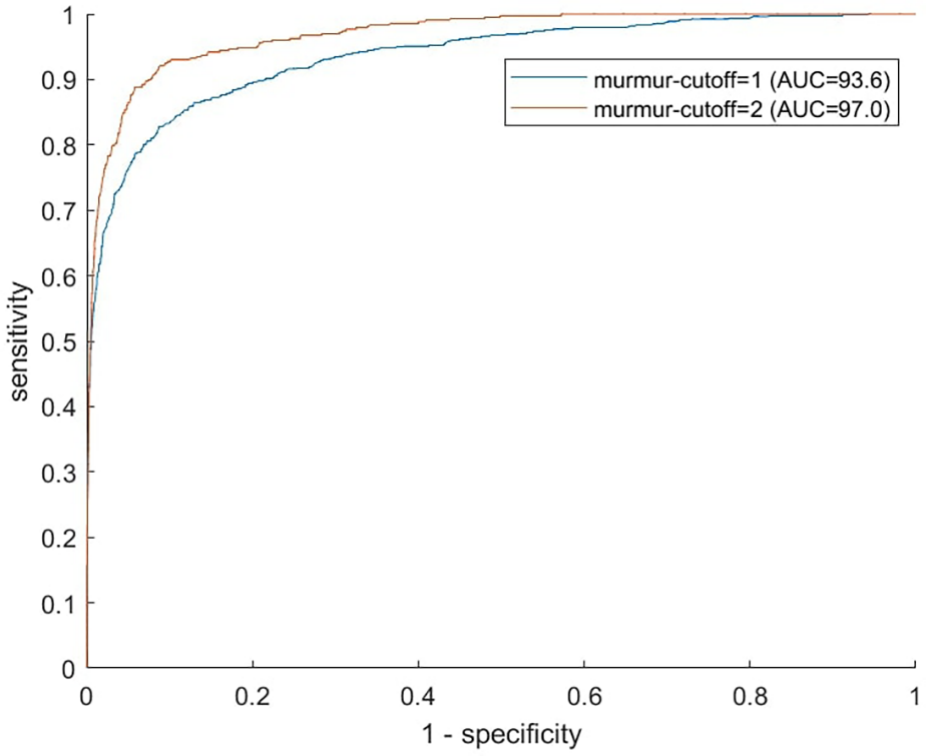
Murmur detection ROC curves. Receiver operator characteristics curves illustrate the algorithm's ability to predict murmur grades ≥1 (blue) and ≥2 (red). Recordings from all four auscultation positions were used in producing the figure.

The murmur detection algorithm detected murmur grade ≥ 2 with an AUC of 0.969 (CI: 0.958–0.982), a sensitivity of 86.5% (CI: 83.2%–89.7%), and a specificity of 95.2% (CI: 94.7%–95.7%). For murmur grade ≥ 1, the AUC decreased to 0.936 (CI: 0.931–0.949). See Figure 2 for ROC curves for cutoff values of 1 and 2. Performance metrics for the prediction of murmur grade ≥ 2 for each auscultation position is given in Table 2, which shows that the ability to predict murmurs did not vary notably between positions. The holdout-set AUC for the prediction of murmur grade ≥ 2 was 0.982 (*n* = 55) and 0.935 for the prediction of murmur grade ≥ 1(*n* = 91), which indicates that the cross-validation results are reliable.

**Table 2:** Performance overview of the murmur detection algorithm.

	Murmur grade ≥ 2				VHD			
	Pos. 1	Pos. 2	Pos. 3	Pos. 4	AS ≥ 1	MS ≥ 1	AR ≥ 3	MR ≥ 3
Sensitivity (%)	86.3 (±5.4)	89.9 (±6.9)	88.0 (±7.1)	81.1 (±9.5)	90.9 (±9.6)	83.3 (±23.2)	54.9 (±8.7)	55.6 (±6.3)
Specificity (%)	92.4 (±1.3)	96.1 (±0.9)	95.6 (±1.0)	96.6 (±9.5)	94.1 (±1.1)	85.7 (±1.6)	65.1 (±2.3)	50.6 (±2.5)
AUC (%)	96.0 (±2.2)	97.3 (±1.6)	95.2 (±0.7)	96.9 (±0.9)	97.9 (±1.6)	92.2 (±6.5)	63.4 (±6.9)	54.9 (±4.4)

Sensitivity, specificity, and area under the curve (AUC) for the prediction of various targets (after dichotomizing) using the murmur prediction algorithm. Note that the murmur prediction algorithm outputs continuous positive values. Values in the AS column were obtained using the multiposition model (described in the Methods section), while the other VHDs were predicted using the highest predicted murmur grade across the auscultation positions. 95% confidence intervals are indicated in the parenthesis.

VHD, valvular heart disease.

### VHD prediction using the murmur detection algorithm

The ability the of murmur detection algorithm to predict clinically relevant VHD is summarized in Table 3, which shows the AUC for predicting each disease for different disease cutoff values. Supplementary Figure S4 shows ROC curves for algorithm prediction of each VHD. AR ≥ 3 was not reliably detected by the algorithm, regardless of which position was used, with AUC values ranging from 0.576 (mitral position) to 0.634 (using the highest predicted murmur grade across positions as a predictor). Symptomatic AR was significantly more prevalent in the detected cases than in the missed cases: 19.4% and 4.9% of cases, respectively (*p* = 0.01). When the positive class was defined as AR ≥ 3 with symptoms, sensitivity increased from 54.9% (±8.7%) to 82.4% (±19.8%; 14 of 17 cases detected), and the AUC for this class was 0.756 (calculated for all CV predictions jointly since some CV validation sets had 0 cases).

**Table 3:** AUC for detecting VHD with predicted murmur grades in each auscultation position.

	AR		MR		AS		MS
	Grade ≥ 3	Grade ≥ 4	Grade ≥ 3	Grade ≥ 4	Grade ≥ 1	Grade ≥ 2	Grade ≥ 1
With pos. 1	**62.2** (±6.8)	**68.1** (±5.1)	52.5 (±4.1)	57.3 (±8.1)	96.7 (±1.9)	98.4 (±2.1)	89.0 (±10.9)
With pos. 2	59.6 (±8.0)	62.1 (±11.3)	**56.3** (±3.1)	57.6 (±13.5)	**98.2** (±1.0)	**98.7** (±0.5)	**89.6** (±8.8)
With pos. 3	58.4 (±6.8)	58.7 (±7.6)	54.9 (±3.3)	**61.3** (±10.4)	97.0 (±1.5)	98.4 (±0.8)	85.4 (±17.6)
With pos. 4	57.6 (±6.0)	56.1 (±10.7)	55.8 (±3.9)	55.8 (±11.0)	92.1 (±4.5)	97.6 (±1.5)	86.0 (±15.5)
Prediction based on all positions^a^	63.4 (±6.9)	67.3 (±8.7)	54.9 (±4.4)	57.8 (±6.9)	97.9 (±1.4)	99.3 (±0.4)	92.7 (±7.8)

AUC of the murmur detection algorithm for predicting various VHD subcategories (defined by valve affected and threshold/grade defining significant cases) using algorithm-predicted murmur grade for the aortic, pulmonic, tricuspid, or mitral position, in that order. The bold values indicate the positions associated with the highest AUC in each column (disregarding values in the last row).

VHD, valvular heart disease; AR, aortic regurgitation; MR, mitral regurgitation; AS, aortic stenosis; MS, mitral stenosis.

^a^ The last row shows values obtained using the highest murmur grade across positions as aggregate prediction, except for AS, which was predicted using the multiposition model that converts the set of predicted murmur grades into predictions of the aortic valve pressure gradient through a regression model.

The AUC for predicting symptomatic cases was higher for AR and MR: 0.756 and 0.711, respectively.

MR was the least detected disease, as MR ≥ 3 was predicted with an AUC of only 0.558 (CI: 0.506–0.593; mitral recording). No consistent trends with regard to which position was most predictive of MR were observed. As was the case for AR, the detectability of MR was highly influenced by the presence of symptoms. Symptomatic MR was significantly more prevalent in the detected cases than in the missed cases: 14.8% and 5.6% of cases, respectively (*p* = 0.02). The algorithm detected 69.2% (±18.7%; 18 of 26 cases) of symptomatic cases, compared to 55.6% (±6.3%) for predicting all MR ≥ 3 cases, and the AUC for these cases was 0.711 (calculated for all CV predictions jointly).

MS ≥ 1 was predicted with a sensitivity of 83.3% (CI: 51.6%–98.0%), a specificity of 85.7% (CI: 84.0%–87.2%), and an AUC of 0.922 (CI: 0.858–0.987). None of the MS recordings had diastolic murmurs, despite the characteristic MS murmur occurring during diastole. Seven of the total 13 cases (53.9%) of MS also had AS, indicating a considerable disease overlap. Of the 10 detected MS cases, four had no presence of AS and three were not associated with any other VHD (excluding grade 1 or 2 AR and MR, which were common in the dataset).

AS ≥ 1 was predicted by the multiposition model with an AUC of 0.979 (CI: 0.963–0.995), a sensitivity of 90.9% (CI: 78.3–97.5%), and a specificity of 94.5% (CI: 93.3–95.5%), whereas AS ≥ 2 was predicted with an AUC of 0.993 (CI: 0.972–0.996). By comparison, the maximum (predicted) murmur grade predicted AS ≥ 1 with an AUC of 0.972 and AS ≥ 2 with an AUC of 0.991. Accuracy was in general higher for more severe AS, as can be seen in Table 3 or Figure 6, which shows the AUC as a function of the AVPGmean cutoff threshold. A positive test using the multiposition model increased the risk of AS ≥ 1 from 2.12% to 19.1%. For AS ≥ 2, the risk increased from 1.27% and 17.1%. A negative result was estimated to rule out the presence of AS with >99% probability.

The pulmonic position was the position that produced the most accurate prediction of both AS ≥ 1 and AS ≥ 2 (see Table 3 for the prediction performance associated with each position), with an associated AUC value of 0.982 (±0.010) for the detection of AS ≥ 1. In contrast, prediction based on the aortic position achieved a lower AUC of 0.967, although the difference was not significant (*p* = 0.16). Screening for AS ≥ 1 on the holdout set using the multiposition model, all six cases were detected, the specificity was 90.6%, and the AUC was 0.988.

Using the multiposition model to predict AS ≥ 1 resulted in four missed cases (of 45 cases), of which three were mild and one was moderate (AVPGmean = 27.17 mm Hg). There were 102 false positive predictions, but of these cases, 20.1% of these observations had AVPGmean > 10 mm Hg.

### Joint screening for clinically relevant cases

**Figure 3:**
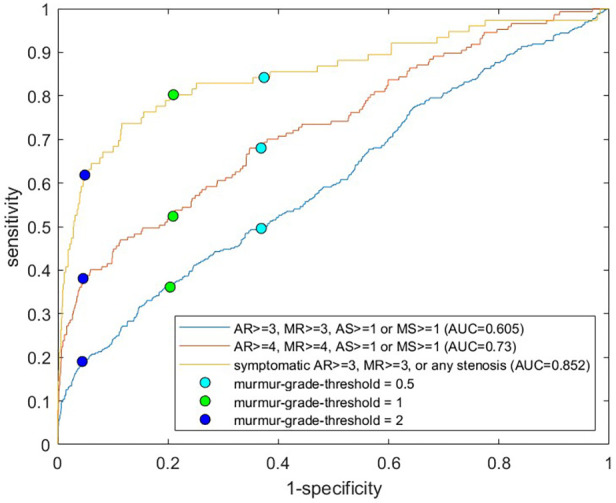
ROC curves for prediction of clinically relevant cases. Each receiver operating characteristics curve corresponds to prediction of a different definition of clinically relevant VHD, with definitions shown in the legend. For the yellow curve, asymptomatic cases of regurgitation were excluded from the positive class. The circles show sensitivities and specificities for murmur grade decision thresholds of 0.5, 1.0, and 2.0. The position with the highest algorithm-predicted murmur grade was used as the predictor. VHD, valvular heart disease; AR, aortic regurgitation; MR, mitral regurgitation; AS, aortic stenosis; MS, mitral stenosis.

**Figure 4:**
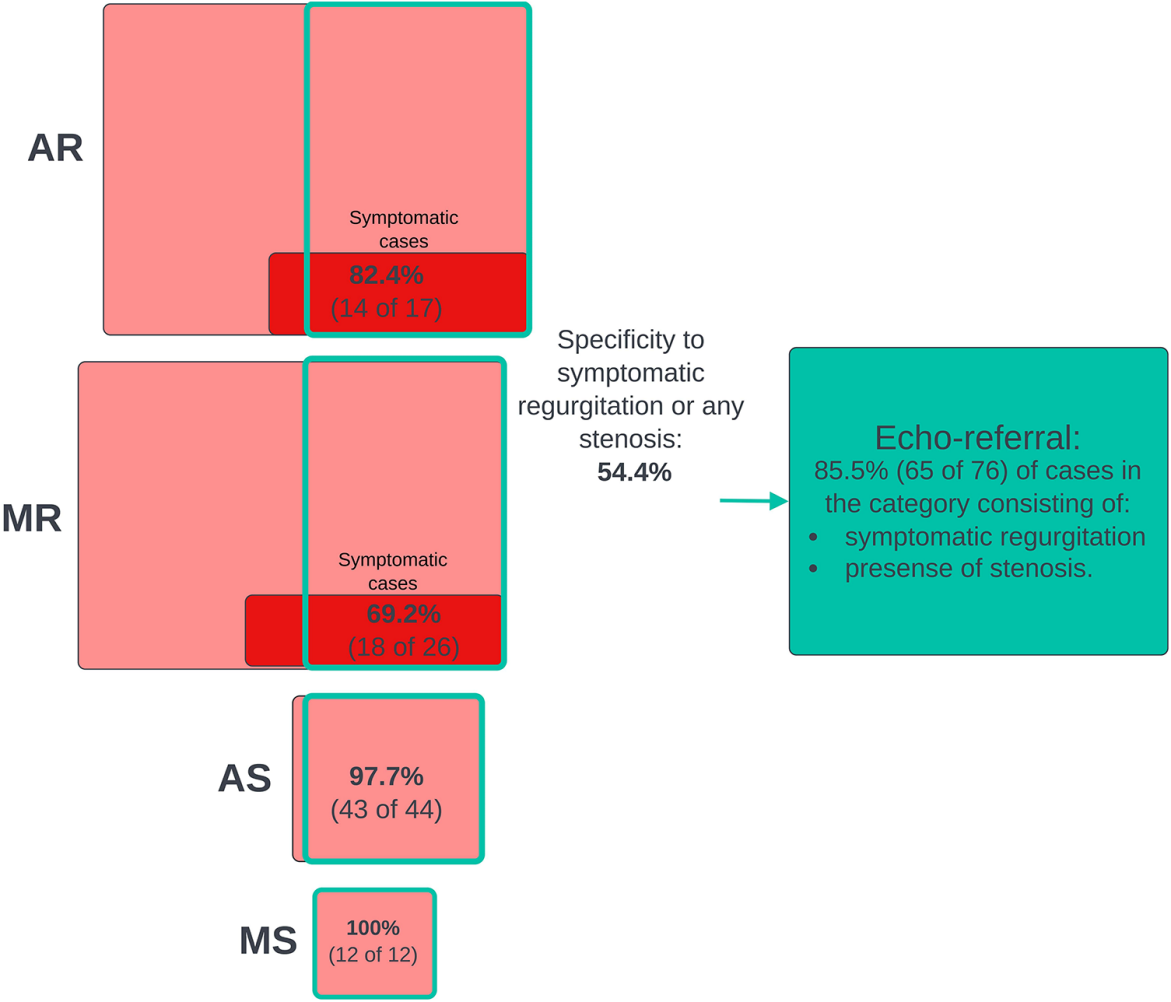
Joint screening for clinically relevant cases. The diagram that shows numbers and proportions of clinically significant cases detected in the process of screening jointly for all clinical cases using the murmur detection algorithm and the prevalence of false positive predictions. The highest algorithm-predicted murmur grade was used as the predictor of clinical cases. A positive prediction was counted as a true positive if the participant had a significant grade for at least one VHD. Included in the pathological VHD categories are grade ≥ 1 stenosis and AS ≥ 1 regurgitation. The red boxes represent subgroups of participants who had or used to have symptoms (shortness of breath while walking calmly on a flat surface or resting). VHD, valvular heart disease; AR, aortic regurgitation; MR, mitral regurgitation; AS, aortic stenosis; MS, mitral stenosis.

We screened for clinically relevant VHD using three different definitions based on different severity thresholds and consideration of symptoms**.** In general, performance metrics increased when the criteria for “clinically significant” was made stricter, either by considering only symptomatic cases of regurgitation as significant or by increasing the AR and MR thresholds. Figure 3 shows the ROC curves for the prediction of the three different clinical categories (outlined in the Methods section), and Figure 4 shows the proportions of important clinical subgroups that were identified. Participants with grade ≥ 3 regurgitation or grade ≥ 1 stenosis were detected with a sensitivity of 56.3%, a specificity of 54.9%, and an AUC of 0.60 (CI: 0.55–0.66). In the process, the algorithm detected 97.7% (43 of 44) of AS cases, all 12 MS cases, 62.4% of AR cases, and 49.2% of MR cases. Screening for grade ≥ 4 regurgitation or grade ≥ 1 stenosis, the algorithm achieved an AUC of 0.73 (CI: 0.66–0.80) and detected 68.9% and 55.4% of AR and MR cases, respectively. Screening for symptomatic grade ≥ 3 regurgitation or grade ≥ 1 stenosis, the AUC was 0.86 (CI: 0.82–0.90) and 65 of 76 cases were detected. When screening for grade ≥ 3 regurgitation or grade ≥ 1 stenosis on the holdout set, the algorithm detected all six cases of the presence of AS, one of one case of MS, nine of 15 cases of AR, and 15 of 36 cases MR and achieved an AUC of 0.652 for overall detection of significant cases.

### Multiposition prediction of the aortic valve mean pressure gradient

The multiposition linear regression model for the prediction of the AVPGmean obtained after model selection and parameter estimation was

$$\mathrm{AVPGmean}=3.5+0.6\cdot MG_{A}^{2}+1.1\cdot MG_{P}^{2}+0.5\cdot MG_{T}+\ldots$$

$$\ldots +0.9\cdot MG_{A}^{2}\cdot \mathrm{nois}e_{P}+8.9\cdot MG_{M}\cdot \mathrm{nois}e_{A}\cdot \mathrm{nois}e_{P}\cdot \mathrm{nois}e_{T}.$$

Subindices A,P,T,andM refers to the aortic, pulmonic, tricuspid, and mitral positions, respectively, $MG_{i}$ denotes the predicted murmur grade of position *i*, and $\mathrm{nois}e_{i}$ is the indicator variable that modifies the murmur grade weights if the recording from position *i* is noisy (in which case $MG_{i}$ is set to zero). The *p*-values for all model parameters were lower than 0.01.

**Figure 5:**
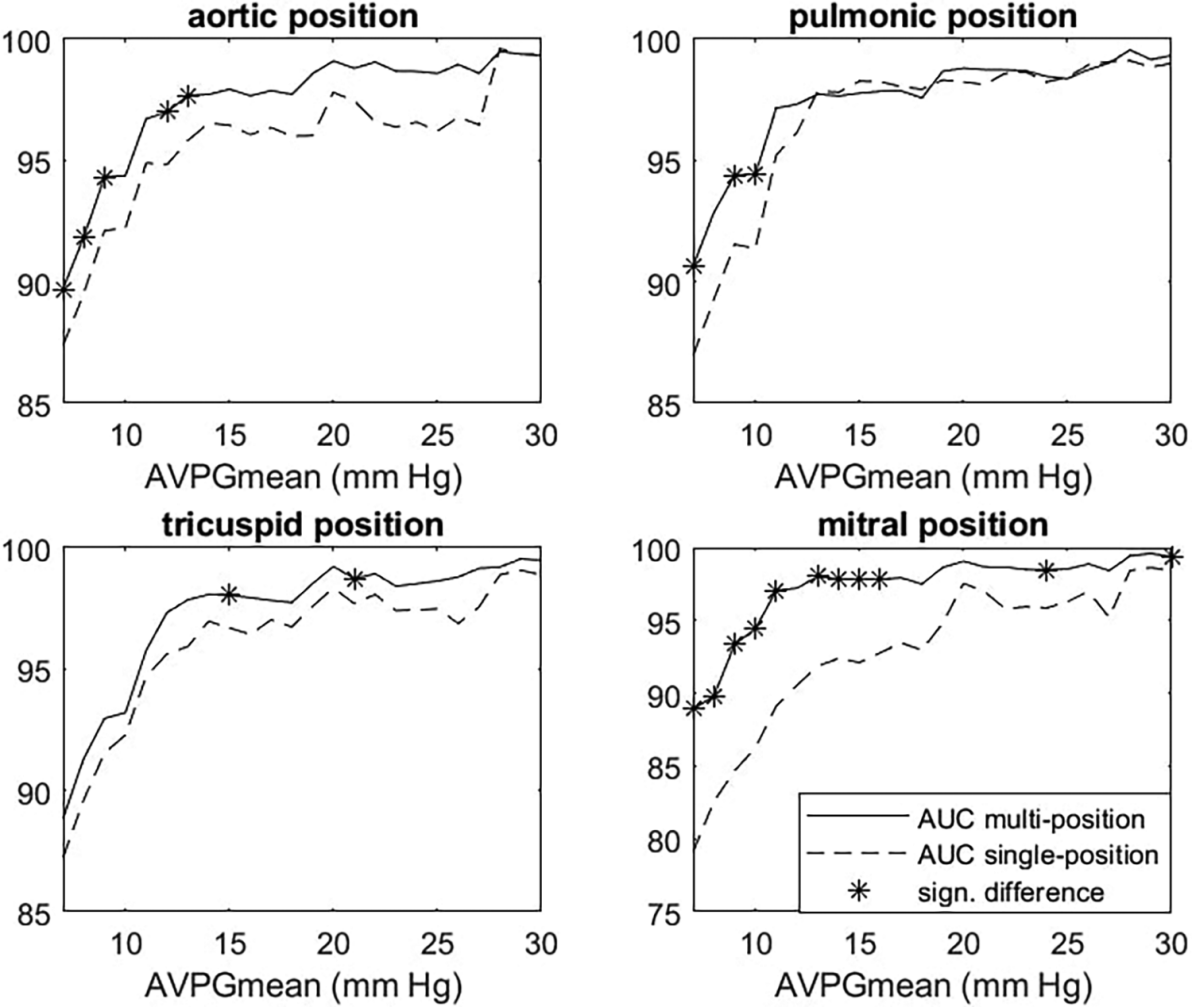
Multiposition model vs. single-position AUC for AVPGmean prediction. Each panel shows AUC for the prediction of cases where AVPGmean > *u* for thresholds *u* ranging from 7 to 30 mm Hg for the single-position (dashed line) and multiposition (solid line) model. Each single-position model uses only data from the index position to make predictions, whereas the multiposition model uses data from all four positions. Significant differences are marked with *. AUC, area under the curve; AVPGmean, aortic valve mean pressure gradient.

After selecting the model, we performed cross-validation to estimate how well the multiposition model predicted the high AVPGmean compared to the single-position models across a range of cutoff thresholds using AUC for the prediction of AVPGmean ≥ threshold as comparison metrics. Figure 5 shows the results for each position, with significant differences indicated by *. For AVPGmean cutoff = 10 mm Hg, the multiposition prediction outperforms each of the single-position predictions by amounts corresponding to *p*-values of 0.09, 0.02, 0.40, and 0.01, respectively.

### VHD prediction with clinical variables

An overview of the multivariate logistic regression models is provided in Table 4, which also shows the AUCs for the prediction of each VHD (as defined by severity grade cutoff values) obtained using cross-validation. The estimated coefficients of each model are listed in Table 5. SpO_2_, smoking, and diabetes were therefore not taken into account as their impacts were not significant.

**Table 4:** Multivariate regression models that include clinical variables.

Prediction target	AUC	Model
AR ≥ 3	0.747 (±0.035)	AR∼murmur grade + age + gender + breathless uphill^a^ + heart rate
MR ≥ 3	0.672 (±0.039)	MR∼murmur grade + age + heart rate
AS ≥ 1	0.982 (±0.019)	AS∼murmur grade + gender + (gender: murmur grade)
MS ≥ 1	0.938 (±0.041)	MS∼murmur grade + age + heart rate

The table shows the variables that were included in the logistic regression models that use clinical variables and predicted murmur grades to predict VHD and the AUC for each model obtained in cross-validation. Interaction between variables is denoted by a “:” symbol. As auscultation-based prediction input, the AR and MS models use the maximum murmur grade, the MR model uses the mitral position murmur grade, and the AS model uses the predicted AVPGmean.

VHD, valvular heart disease; AR, aortic regurgitation; MR, mitral regurgitation; AS, aortic stenosis; MS, mitral stenosis.

^a^ Breathless when walking rapidly on level ground or up a moderate slope.

**Table 5:** Estimated parameters of clinical variable models.

Prediction target of model	Age	Murmur grade	Murmur grade : gender (interaction)	Gender	Breathless	Heart rate
AR ≥ 3	0.0034***	0.0390***	—	0.0330*	0.0270*	−0.0017**
MR ≥ 3	0.0052***	0.0120	—	—	—	−0.0033***
AS ≥ 1	—	0.0247***	0.0248***	−0.0848***	—	—
MS ≥ 1	0.0004*	0.0200***	—	—	—	0.0008***

The table shows the estimated coefficients of the variables included in the multivariable logistic regression models. The *Murmur grade* column shows the effect of the explanatory variable derived from murmur grade predictions; the AR and MS models use the predicted maximum murmur grade, the MR model uses the mitral position predicted murmur grade, and the AS model uses the output from the multiposition model. Reference category for gender is female. Interaction between variables is denoted by the “:” symbol. Statistical significance of the estimated parameters is denoted by (*), (**), and (***), which indicates *p*-values within intervals (0.01–0.05), (0.001–0.01), and (0–0.001), respectively.

VHD, valvular heart disease; AR, aortic regurgitation; MR, mitral regurgitation; AS, aortic stenosis; MS, mitral stenosis.

Inclusion of clinical variables in addition to predicted murmur grades improved screening performance significantly for both AR (AUC from 63.4 to 0.747, *p* = 0.002) and MR (AUC from 55.8 to 0.672, *p* = 0.007). The prediction of both AS ≥ 1 and MS ≥ 1 was improved but not significantly, with AUCs increasing from 0.979 to 0.982 (*p* = 0.30) and from 0.922 to 0.938 (*p* = 0.33), respectively. AS ≥ 2 was detected with an AUC of 0.995 (CI: 0.991–0.998, up from 0.993, *p* = 0.17), a sensitivity of 96.3% (CI: 81.0%–96.3%), and a specificity of 96.7% (CI: 95.7%–97.4%). AS ≥ 1 was detected with a sensitivity of 88.6% and a specificity of 95.0%. Of the false positives, 35.9% had either at least moderate AR, at least moderate MR, or AVPGmean > 10 mm Hg.

In all models except the AS model, age and heart rate were significant predictors. The interaction between gender and murmur grade was highly significant (*p*-value < 0.0001).

## Discussion

We trained an RNN to detect and grade heart murmurs in heart sound audio and used these predicted grades to predict clinical VHD. We demonstrated for the first time that even mild AS can be predicted accurately by ML algorithms from heart sounds in a general population. AR and MR were poorly predicted from algorithm-predicted murmurs but accuracy increased significantly when only symptomatic cases were considered significant. We also found evidence that utilizing heart sound recordings from multiple auscultation positions can be beneficial for the prediction of the mean aortic valve pressure gradient. Finally, we used the RNN and multivariate logistic regression to combine cardiac auscultation and clinical data and showed that incorporation of both kinds of data is highly beneficial in screening for AR and MR. The improvement in AS and MS prediction was not quite significant, but the highly significant *p*-values of the coefficients indicated that this could be due to an underpowered dataset. The adjusted association between the murmur grade and AS was stronger for men than for women.

### Comparison with clinicians

**Figure 6:**
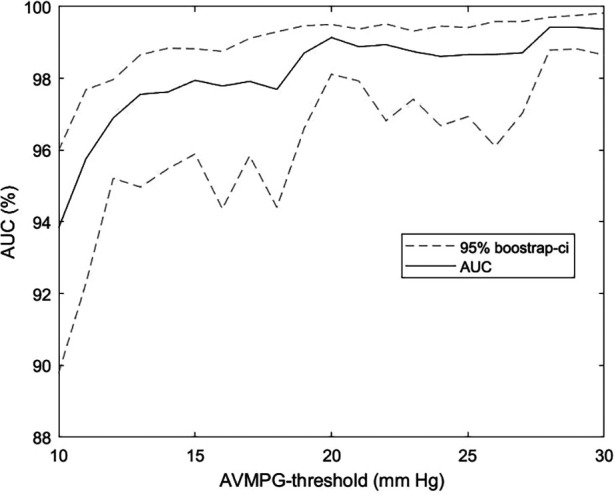
AUC as a function of the aortic stenosis cutoff threshold. AUC for the prediction of AVPGmean > *u* across a range of thresholds *u* using the multiposition model on the subset with noise in up to three out of four auscultation positions. Confidence intervals were obtained using bootstrapping. Recordings annotated as noisy in all four positions were excluded from the analysis. AVPGmean, aortic valve mean pressure gradient; AUC, area under the curve.

A sensitivity of 90.9% and a specificity of 94.5% for detecting AS ≥ 1 compare favorably against metrics on clinician accuracy we found in the literature. In a study by Jaffe et al. (28), severe AS was detected by clinicians with a considerably lower sensitivity of 83% and specificity of 79%. In a 2021 study by Chorba et al. (29), three expert cardiologists classified recordings primarily from the aortic position into the presence or absence of murmur, and these classifications were subsequently used to predict moderate to severe AS in a selected cohort of confirmed pathological cases (moderate to severe) and healthy controls. The highest performance (in terms of sensitivity + specificity) achieved via this prediction scheme was a sensitivity of 82.5% (CI: 69.6%–93.6%) and a specificity of 90.2% (CI: 83.1%–-96.3%), and the average sensitivity and specificity across annotators were 90.0% and 71.1%, respectively, which our algorithm outperformed by a considerable margin. Note that the sensitivity and specificity for AS ≥ 2 were not computed in cross-validation because one of the eight validation sets ended up not containing any cases, as stratification ensured only an even distribution of AS ≥ 1. As can be seen in Figure 6, there is a clear trend for detectability to increase with higher pressure gradient thresholds, so the sensitivity and specificity of our models would likely be higher for moderate or severe AS.

### Comparison with the literature on automated HS analysis

We found only two studies on automated HS analysis that could be meaningfully compared to our study. Aside from these studies, the performance metrics for specific VHDs were not presented or the data that were used had too little noise to be reasonably representative of a day-to-day clinical setting. Of the literature we identified, our study presented the highest accuracy for the prediction of AS. Makimoto et al. predicted severe AS with an AUC of 0.968 when training directly on AS using convolutional neural networks and three auscultation positions (30). The study by Chorba et al. (29) offered an interesting comparison, as their methodology is very similar to ours. They trained a deep convolutional neural network to predict murmur grades, achieving an AUC of 0.958 for predicting murmur grade ≥ 2, which we outperformed with an AUC of 0.969 (±0.12). Furthermore, they achieved 93.2% sensitivity (CI: 86.9%–98.5%), 86.0% specificity (CI: 80.9%–91.0%), and 0.952 AUC for the prediction of moderate to severe AS based preferentially on the aortic recording, which we exceeded with an AUC of 0.967 when using the aortic recording for prediction. We improved the prediction of AS further by combining all four algorithm-predicted murmurs in a multiposition model, obtaining AUC values of 0.978 (CI: 0.961–0.994) and 0.992 (CI: 0.988–0.997) for the prediction of at least mild and at least moderate AS, respectively. We note that Chorba et al. preferred using the aortic recording (which is often recommended for detecting the AS murmur) for the prediction of AS using the pulmonic position as a secondary choice, yet our results and those of Makimoto et al. suggest that the pulmonic recording might be more suitable for detecting AS.

A caveat to the above comparison is that Chorba et al. and Makimoto et al. referenced existing guidelines for echocardiography but did not describe in full detail how they graded AS. Thus, differences in performance could be due to differences in the AS-grading convention. In particular, defining AS in terms of the AVPGmean could result in a target that is easier to predict, as it may exclude cases where the pressure gradient is low due to low cardiac output, which might be harder to detect. However, a 2012 study tracking the progression of AS in a cohort representative of the general Norwegian population found that accounting for systolic left ventricular dysfunction after initial grading based on the AVPGmean did not change the classification of any participants. We therefore believed it is unlikely that we missed a significant number of cases (13). Differences in the prevalence of “low-flow low-gradient” cases cannot be disregarded as a source of performance difference, however.

The higher AS and murmur detection rates discussed above might in part be attributable to the particularly rigorous annotation procedure that produced our murmur annotations, resulting in a Cohen's kappa for agreement on the presence of murmur grade ≥ 1 of 0.717 and agreement on the presence of any murmur in 94.2% of recordings. In contrast, assessing cardiologist agreement on the presence of any murmur, a study by Andersen et al. found a median Cohen's kappa of 0.69 and a mean proportion of interrater agreement of 84% (from pairwise comparisons), with lower values for GPs and medical students (11). Chorba et al. reported a Fleiss kappa of 0.478 (three cardiologist raters), which is lower than the Fleiss kappa of 0.69 observed by Andersen et al. for their eight cardiologist raters. Thus, it is likely that our raters had substantially higher inter-rater agreement than that seen in Chorba et al.'s study.

### Literature comparison for MR detection

While we detected AS and murmurs more accurately than in Chorba et al.'s study, our results for detection of at least moderate MR were dramatically worse, as they predicted these cases with an AUC of 0.865 (29 cases, 62 healthy controls), while our prediction was barely better than chance. Given that we trained our algorithms to predict the same target (murmur) and achieved very similar performances in murmur detection, this wide performance gap cannot be explained by differences in model performance, suggesting that significant MR in cohorts consisting of patients and healthy controls is more discernible than in unselected cohorts. Consistent with this observation is the fact that detection rates for both AR and MR increased significantly within our study when only symptomatic instances of diseases were treated as prediction targets. Supporting this conclusion further is a 2018 study by Gardezi et al. (9) in which they tested the accuracy of auscultation in a population of asymptomatic patients aged 65 > years and found that a diagnosis of significant VHD by auscultation was not significantly better than chance. Myerson et al. found similar results in their 2017 study of asymptomatic participants aged ≥ 65 years with no previous VHD diagnosis, where GPs identified significant VHD (moderate to severe regurgitation or at least mild stenosis) with an AUC of only 0.56 (31). Taking all of this into account, it seems likely that the observed poor performance for detecting MR may be due to a lack of clearly discernible disease features in the heart sounds and not poor algorithm performance. It is also possible that murmurs alone simply are not reliable predictors of MR in general populations and that reasonable performance could be achieved if models were instead trained directly on MR, assuming a dataset with sufficiently many examples. The inclusion of simple clinical variables yielded considerable boosts in accuracy, and we suspected that exploring models that incorporate a variety of data sources might be a more fruitful approach when it comes to screening for AR and MR in a general population.

### Optimal utilization of positions for AS predictions

The multiposition model seems the overall preferred model for predicting AS, as it generally outperformed the single-position-based predictions. This conclusion is supported by research of Makimoto et al., who also found that using multiple positions improved the prediction of AS (30). We compared models on their ability to separate high from low AVPGmean (measured by AUC) and varied the threshold defining the “high” category. Comparing it to the position with the highest AS detection accuracy, the pulmonic position, it performed equally well for discerning very high pressure gradients (separation thresholds between 15 and 30 mm Hg) but was significantly more accurate at distinguishing AVPGmean in the normal range from only slightly elevated AVPGmean (separation thresholds between 7 and 10 mm Hg). More accurate identification of slightly elevated pressure gradients might be of clinical interest, as a general cohort study by Eveborn et al. found that the group of participants with 10 ≤ AVPGmean < 15 mm Hg at baseline identified about half of those who developed AS in the following 7 years (32). Another advantage of the multiposition model is that it is not limited to cases with clean pulmonic or aortic recording; we have demonstrated its high accuracy on a set that includes samples with noisy recordings in up to three positions. Although the AUC analysis was not entirely consistent in terms of statistical significance, the highly significant *p*-values (<0.01) of the fitted model parameters suggested that all positions contribute significantly to the prediction of the pressure gradient, and we therefore suspected that having more cases of AS would show a significant benefit to AS detection. Note that some of the detected AS murmurs might have been caused by MR due to disease overlap, but given how strongly AS was related to murmurs relative to MR, this overlap seems unlikely to explain a significant number of AS murmurs, and a positive prediction should be followed up with an echocardiographic examination in any case.

### Clinical implications

Our results clearly demonstrate the high potential of machine learning algorithms for AS screening in a general population. A digital stethoscope that automatically analyzes the recorded audio using our algorithms could serve as a low-cost, easy-to-use, accurate, and highly available screening tool for detecting AS, and its implementation into routine clinical practice could substantially reduce costs from unnecessary echocardiography examinations. The need for such a tool was demonstrated by a study by d'Arcy et al., who found a 1.3% prevalence of newly diagnosed AS in a general population of individuals aged ≥ 65 years (33). Automated HS analysis also creates opportunities for remote patient monitoring, meaning that heart sounds can be monitored frequently and consistently without subjecting vulnerable patients to the risks and challenges associated with hospital visits.

Another important implication of our study is the possibility of detecting AS in the general population before symptoms become apparent, and various studies indicate that our algorithms could be especially useful for identifying a subset of this group who are likely to benefit from close monitoring or aortic valve surgery prior to the onset of symptoms. Rosenhek et al. found that a peak aortic jet velocity ≥ 5.5 m/s in patients with very severe asymptomatic AS was associated with a very poor prognosis (event-free survival rate ≤ 44  ∓ 8% at 1 year, where events were aortic valve surgery or death), and the peak aortic jet velocity was in general a strong predictor of the rate of event-free survival (18). Rosenhek et al. and Otto et al. found similar results in their studies on asymptomatic AS and, in addition, observed that a rapid increase in jet velocity was an independent predictor of clinical outcomes (aortic valve surgery or death) in a multivariate analysis (19, 34). Since the jet velocity is closely related to the pressure gradient, our models could potentially be used to detect a high rate of change in jet velocity.

To put the screening performance of our algorithms into perspective, we assumed a 1.3% prevalence of undiagnosed AS in those aged ≥ 65 years (assuming that ours and their definition of the presence of AS matches reasonably well) and estimated that given a population size of 10,000 elderly individuals, our multivariate risk-factor algorithm would detect 115 of the expected 130 individuals with undiagnosed mild or greater AS, at the cost of producing ∼495 false positives. If we reclassify 35.9% of false positives due to the presence of other VHDs or AVPGmean > 10 mm Hg (32) (see the section on the clinical factor models), the expected number of false positives decreases to 178. To our knowledge, no existing low-cost procedure is capable of providing such screening performance. Point-of-care ultrasound has been launched as a potential solution, but it will take many years before most GPs are fully trained in this; studies show no improvement in AS detection over auscultation (35–37), and adding this procedure to the physical examination will not help with the time constraints on healthcare professionals.

Beyond diagnosing and monitoring AS, there are data to suggest that a murmur detection algorithm could help identify high-risk subgroups that are not typically considered for auscultation. A recent 35-year follow-up study demonstrated that murmur grade 2 or higher in seemingly healthy middle-aged men was associated with an 89.3-fold (CI: 39.2–211.2) age-adjusted risk of undergoing aortic valve replacement later in life and a 1.5-fold (CI: 0.8–2.5) age-adjusted increased risk of CVD death (38). Thus, even in subgroups not typically considered for cardiac auscultation, screening for murmurs could provide clinically valuable information.

An interesting finding in the multivariate analysis of predicted heart sounds and risk factors was a highly significant (*p* < 0.0001) positive interaction between the predicted murmur grade and male gender in predicting AS risk, indicating that a unit increase in the predicted murmur grade for men is associated with a larger increase in AS risk than it is for women. In terms of unadjusted risk ratios, we found that men with a mean (human annotated) murmur grade ≥ 2 had a 126 times greater risk of AS compared to men with murmur grade < 2, whereas the corresponding value for women was 77. It might therefore be appropriate to take gender into consideration when deciding clinical thresholds for follow-up or incorporate gender into the risk calculations in multivariate models.

### Study and algorithm strengths and limitations

The biggest limitation of our study is that the models have not been tested in external datasets, and we do not know how robust the models are to changes in the environment in which the data are collected. In particular, we do not know how well the AS detection performance will hold in hospital settings and other geographical areas that may have a greater prevalence of low-flow, low-gradient cases. We are currently planning to collect external data with a higher prevalence of VHD to test the algorithm externally and assess whether model performance (in clinical and general populations, respectively) can be improved by training on AR and MR in a dataset that contains more significant cases.

A practical limitation of the murmur algorithm is that it does not automatically detect noise, which means that the user of the algorithm is relied upon to decide which recordings are of sufficient quality. This is not an issue when the user is a clinician, but it could be an issue when the user is a patient performing the procedure at home. The main limitation of our murmur prediction into the VHD prediction approach is that features other than the murmur grade that might help to discern pathologies, such as loudness or softness (absolute or relative) of S1 or S2, cannot be learned and utilized. The accuracy with which the algorithm detects a disease is therefore limited by the degree to which it is associated with the subjectively rated murmur grade.

The other study limitations are related to the small number of VHD cases and a resulting lack of statistical power in various analyses. Decisions on which covariates to include in the clinical-variable models were made on the basis of predicting a relatively small number of pathological cases, which increases the risk of false positive results. The dataset was also arguably underpowered (depending on which metric is considered) for establishing if using multiple position results is more accurate AS prediction than the pulmonic position. We were also limited in terms of model development, as we had to restrict ourselves to exploring a small set of fairly simple models to limit the risk of overfitting. Finally, we note that the recordings used in this study were collected in a more controlled setting than can be expected in a rushed clinical environment, and further work is likely necessary to improve robustness to noise and interference.

A strength of the algorithm is that we have demonstrated its accuracy on a dataset containing samples with noise in up to three positions and that it utilizes information from all four standard positions to improve the performance of state-of-the-art audio segmentation algorithms, which in turn improves murmur detection performance. Only 1.4% of the dataset was excluded from analysis due to all recordings containing noise (except when analyzing prediction of MR from heart sounds, where we used only the mitral position), so the performance metrics we present are unlikely to be substantially biased due to the exclusion of noisy samples. Another strength of the algorithm is the relatively small size of the model used to predict the murmur grade, which entails fast computation and less time required for experimentation. We also believe its size makes it less prone to overfitting than deeper neural networks that are often employed in automated heart sound classification research, as their enhanced flexibility might predispose them to adapt to features that are specific to the development set, such as the type of stethoscope used.

The main novelty and strength of our study lies in the unfiltered nature of the dataset—a unique feature in automated heart sound classification research that offered us the opportunity to get a realistic assessment of the ability of machine learning to screen for undetected VHD. Another feature novel to this field is the simultaneous access to murmur annotation, results from echocardiography, and data on various clinical variables. Finally, the HS annotations used in this study were obtained using a rigorous procedure in which considerable effort was put into ensuring consistent data annotation and high interrater agreement, and we believe this has resulted in particularly high-quality training data which in turn contributes to high algorithm prediction performance.

## Conclusions

In this study, we developed a murmur detection algorithm that detects mild or greater AS in a general population with an accuracy similar to or exceeding previously reported metrics from similar studies that were based on selected cohorts. The prediction accuracy of AS benefited from using a model that utilized audio from all four standard auscultation positions, achieving high accuracy on a dataset that excluded only the samples that had noisy audio in all four positions. AR and MR were not reliably detected, but detection accuracy increased significantly when only symptomatic cases were targeted and when age, gender, heart rate, and dyspnea were included as predictors. Our results indicate that automated HS analysis could be a highly cost-effective screening tool for detecting undiagnosed VHD.

## Data Availability

The data analyzed in this study are subject to the following licenses/restrictions: The Tromsø Study data are not publicly available, but researchers can apply for access. Requests to access these datasets should be directed to https://uit.no/research/tromsostudy. Further enquires can be directed to the corresponding author.
