# Supplementary material for: Algorithm for predicting valvular heart disease from heart sounds in an unselected cohort
Source: Front Cardiovasc Med. 2024 Jan 24;10:1170804. doi: 10.3389/fcvm.2023.1170804 (PMC10847556; doi:10.3389/fcvm.2023.1170804)

# **SUPPLEMENTARY MATERIALS to Algorithm for Predicting Valvular Heart Disease from Heart Sounds in an Unselected Cohort**

##

##


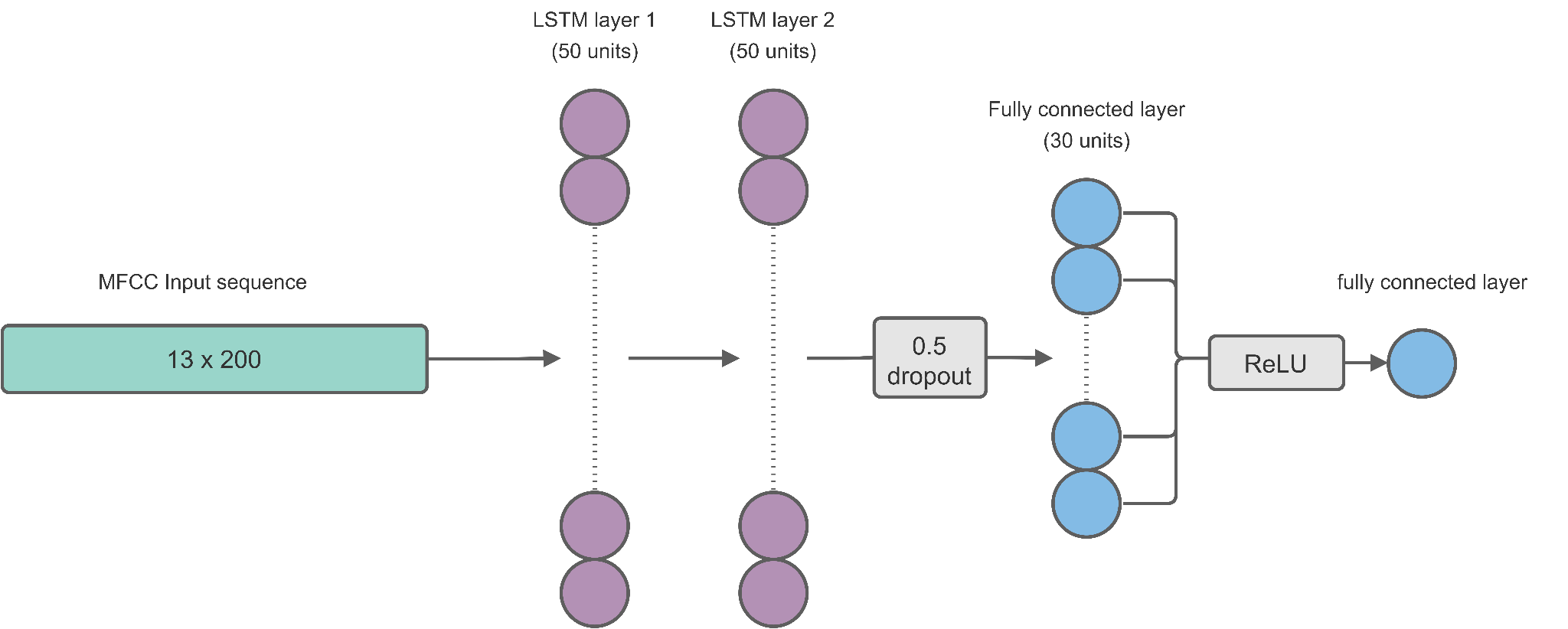


**Figure S1. Network Architecture**


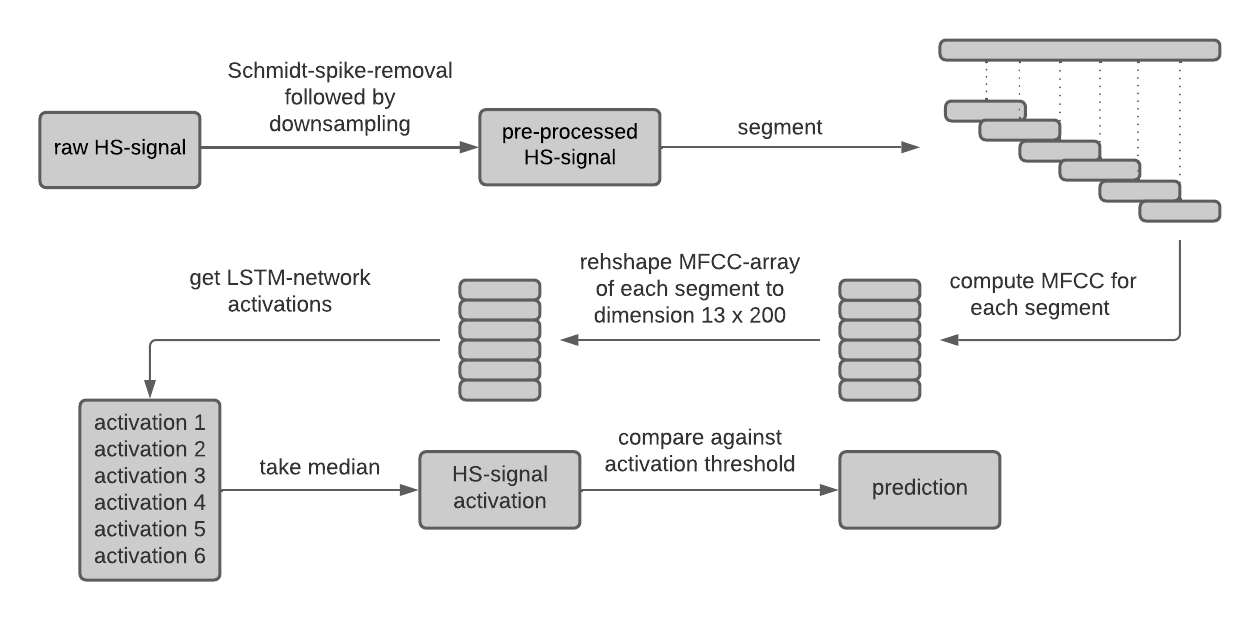


**Figure S2. Processing steps between raw heart sound input and algorithm prediction.**


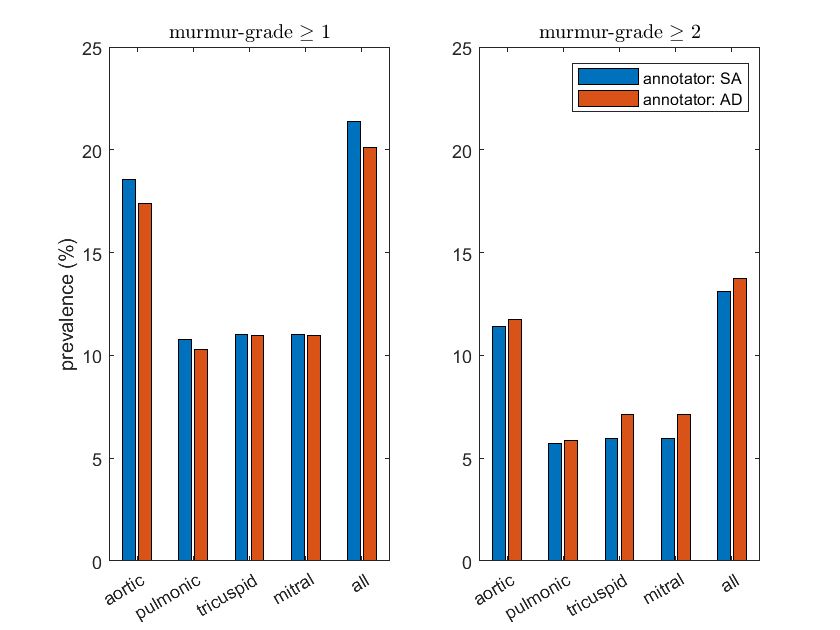


**Figure S3. Prevalence of murmur by recording site.**

The left panel shows the percentage of recordings for which each annotator (annotator names are abbreviated to SA and AD) rated as murmur grade≥1. The panel to the right

shows the same information, but for murmur grade≥2. The bars furthest to the right (labeled “all”) show the percentage of observations for which at least one auscultation position had a murmur grade that exceeded the cutoff threshold.
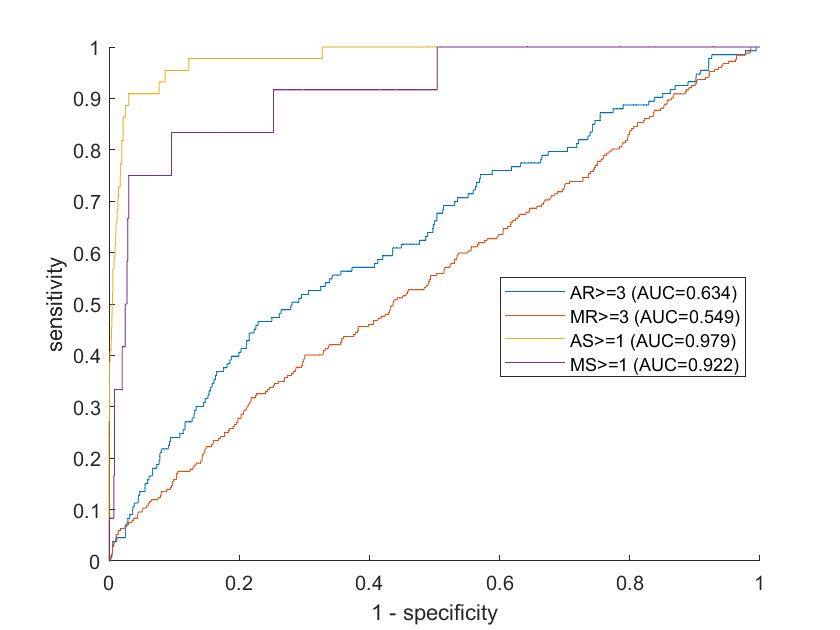


**Figure S4. ROC curves for prediction of each VHD**

##
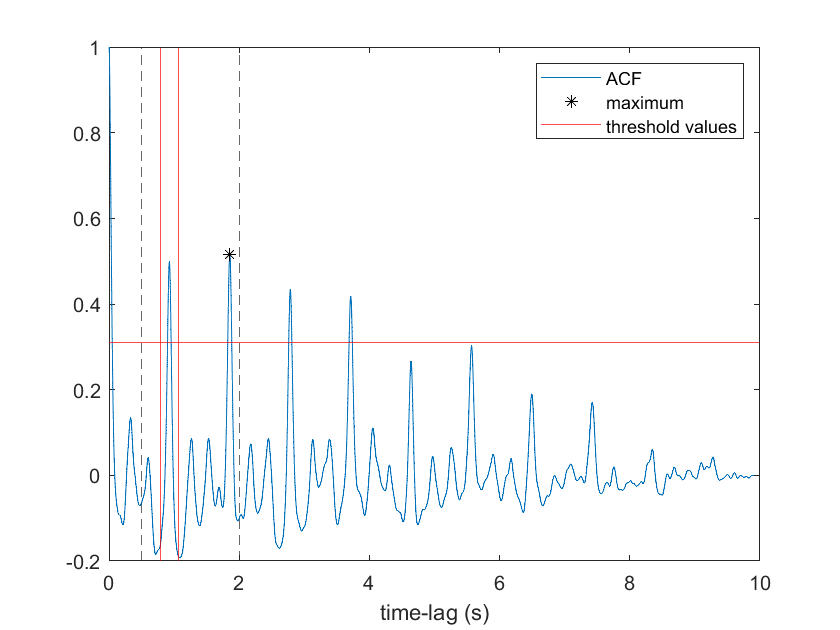


**Figure S5.** **Estimation of heart rate from the autocorrelation function**

Example of how the modified version of Springer's segmentation algorithm identifies the heart rate peak in the autocorrelation function corresponding to the heart sound audio. In this case it inferred that the largest peak (*) represents a multiple of the heart rate peak. Threshold values are calculated based on the largest peak within the search interval [0.5s, 2s].

| **Count of VHD cases [% of Tromsø7 cohort]** | | | | |
| --- | --- | --- | --- | --- |
|  | **AR** | **MR** | **AS** | **MS** |
| **grade≥1** | **461 (21.7%)** | **1078 (50.8%)** | **51 (2.4%)** | **13 (0.612%)** |
| **grade≥2** | **256 (12.1%)** | **805 (37.9%)** | **32 (1.51%)** | **3 (0.141%)** |
| **grade≥3** | **150 (7.06%)** | **292 (13.7%)** | **6 (0.282%)** | **1 (0.0471%)** |
| **grade=4** | **66 (3.1%)** | **69 (3.2%)** | **-** | **-** |
| **Count of symptomatic VHD cases [% of VHD subgroup with symptoms]** | | | | |
|  | **AR≥3** | **MR≥3** | **AS≥1** | **MS≥1** |
|  | **20 (13.4%)** | **33 (11.3%)** | **13 (19.6%)** | **10 (23.1%)** |
| **Demographics within each VHD subgroup** | | | | |
|  | **AR≥3** | **MR≥3** | **AS≥1** | **MS≥1** |
| **female, n** | **66 (46.9%)** | **145 (51.8%)** | **16 (31.4%)** | **10 (76.9%)** |
| **male, n** | **84 (53.1%)** | **147 (48.2%)** | **35 (68.6%)** | **3 (23.1%)** |
| **mean age (SD)** | **71.3 (0.73)** | **69 (0.6)** | **74.9 (1.9)** | **77.7 (1.4)** |
| **40-49, n** | **5 (3.3%)** | **17 (5.8%)** | **0 (0%)** | **0 (0%)** |
| **50-59, n** | **8 (5.3%)** | **33 (11.3%)** | **1 (2.0%)** | **0 (0%)** |
| **60-69, n** | **39 (26%)** | **83 (28.4%)** | **11 (21.6%)** | **2 (15.4%)** |
| **70-79, n** | **73 (48.7%)** | **119 (40.8%)** | **22 (43.1%)** | **5 (38.5%)** |
| **>80, n** | **25 (16.7%)** | **40 (13.7%)** | **17 (33.3%)** | **6 (46.2%)** |
| **Questionnaire and clinical data within each VHD subgroup** | | | | |
|  | **AR≥3** | **MR≥3** | **AS≥1** | **MS≥1** |
| **Dyspnea (while resting or walking on flat surface), n** | **6 (4.3%)** | **8 (2.9%)** | **5 (10.4%)** | **2 (16.7%)** |
| **Angina pectoris (current or previous), n** | **10 (7%)** | **26 (9.5%)** | **9 (20%)** | **0 (0%)** |
| **Diabetes (current or previous), n** | **8 (5.6%)** | **13 (4.6%)** | **5 (10.6%)** | **2 (18.2%)** |
| **Chest pain (current or or previous, while walking up hills or stairs, or fast or normal pace on level ground), n** | **20 (13.3%)** | **37 (13.1%)** | **5 (10.2%)** | **2 (16.7%)** |
| **High blood pressure (current or previous), n** | **72 (49%)** | **114 (40.7%)** | **28 (56%)** | **8 (66.7%)** |
| **Heart rate, mean** | **61 (1.6)** | **61 (1.2)** | **62 (3.2)** | **71.8 (6.2)** |
| **BMI [kg/m^2], mean** | **26.4 (0.7)** | **26.2 (0.5)** | **26.9 (1.2)** | **28 (2.7)** |
| **Smoker (current or previous), n** | **87 (58.4%)** | **165 (57.5%)** | **30 (61.2%)** | **8 (61.5%)** |
| **Missing data within each VHD subgroup** | | | | |
|  | **AR≥3** | **MR≥3** | **AS≥1** | **MS≥1** |
| **Dyspnea missing, n** | **9 (6.0%)** | **16 (5.5%)** | **3 (5.9%)** | **1 (7.7%)** |
| **Angina missing, n** | **8 (5.3%)** | **17 (5.8%)** | **6 (11.8%)** | **4 (30.8%)** |
| **Diabetes, missing n** | **6 (4%)** | **12 (4.1%)** | **4 (7.8%)** | **2 (15.4%)** |
| **Chest pain, missing n** | **0 (0%)** | **10 (3.4%)** | **2 (3.9%)** | **1 (7.7%)** |
| **High blood pressure, missing n** | **3 (2%)** | **12 (4.1%)** | **1 (2%)** | **1 (7.7%)** |
| **Heart rate, missing n** | **5 (3.3%)** | **25 (8.6%)** | **5 (9.8%)** | **1 (7.7%)** |
| **Smoker missing, missing n** | **1 (0.7%)** | **5 (1.7%)** | **2 (3.9%)** | **0 (0%)** |

**Table S1. Summary statistics for study population and dataset variables.**

The first table shows counts and percentages for each VHD within the whole study cohort (2124 participants). The last 3 tables show various statistics within the subcohort indicated by the column header, and percentages represent fractions of those with the indicated VHD (e.g., 31.4% of those with AS≥1 were female). VHD = ventricular heart disease. AR = Aortic regurgitation. MR = mitral regurgitation. AS = aortic stenosis. MS = mitral stenosis. SD = standard deviation. BMI = body mass index.

* Chest pain variable represents chest pain experienced when walking up hills or stairs, or walking fast on level ground, or when walking at normal pace at level ground.

##

|  | **AR** | | **MR** | | **AS** | | **MS** | |
| --- | --- | --- | --- | --- | --- | --- | --- | --- |
|  | **n (%)** | **RR (CI)** | **n (%)** | **RR (CI)** | **n (%)** | **RR (CI)** | **n (%)** | **RR (CI)** |
| **VHD grade ≥1** | **129 (28.0%)** | **1.73 (1.46-2.06)** | **224 (20.8%)** | **1.17 (1.06-1.29)** | **47 (92.2%)** | **52.4 (18.99-145)** | **12 (92.3%)** | **53.52 (6.98-410)** |
| **VHD grade ≥2** | **77 (30.1%)** | **1.92 (1.5-2.45)** | **171 (21.2%)** | **1.2 (1.06-1.37)** | **31 (96.9%)** | **138.3 (18.93-1010)** | **3 (100%)** | **-** |
| **VHD grade ≥3** | **53 (35.3%)** | **2.44 (1.78-3.34)** | **75 (25.7%)** | **1.54 (1.21-1.96)** | **6 (100%)** | **-** | **1 (100%)** | **-** |
| **VHD grade ≥4** | **30 (45.5%)** | **3.72 (2.32-5.96)** | **22 (31.9%)** | **2.09 (1.27-3.42)** | **-** | **-** | **-** | **-** |

**Table S2. Statistical relationship between VHD and murmur grade.**

The table shows the number and percentage of participants with (mean) murmur grade≥1 within the subgroup with the VHD indicated by the row and column. The table also shows risk-ratios (95% CI in parenthesis) associated with presence of murmur, showing how much murmur grade≥1 increases the risk of the indicated VHD. Some risk-ratios could not be computed due to there being no cases in the positive class for which there was absence of murmur, resulting in division by zero. RR = risk ratio. AR = Aortic regurgitation. MR = mitral regurgitation. VHD = ventricular heart disease. AS = aortic stenosis. MS = mitral stenosis. CI = confidence interval.

|  | pos. 1 (aortic) | pos. 2 (pulmonic) | pos. 3 (tricuspid) | pos. 4 (mitral) | all pos. combined |
| --- | --- | --- | --- | --- | --- |
| p-value | 0.4715 | 0.0195* | 0.0488* | 0.0237* | 0.0131* |

**Table S3.** **Performance comparison: regression vs classification**

The table shows the results from a comparison between a network trained to predict murmur grade≥2, and a network trained to predict murmur grade as a continuous variable. The p-values correspond to the hypothesis that the AUC of the regression model is higher than the classification model trained on a binary output (murmur grade≥2). Each column shows the result when predictions are made on audio from the index position. For the rightmost column, the algorithms made predictions on audio from all 4 auscultation positions, and the combined AUC was considered.

## Training with VHD as label

In attempting to develop a model to predict VHD, we considered two possible approaches: 1. training on a proxy target which is more numerous but less clinically relevant (murmurs), or 2. training directly on the target of interest, i.e., VHD. Training the network on disease labels directly can allow the networks to detect more complex disease features, but can also be infeasible since there are so few cases to train on. Training the networks to differentiate moderate to severe AR directly failed to improve prediction significantly beyond what was achieved with the murmur detection algorithm, as it predicted moderate to severe AR with an AUC of only 55.9% (CI:45.4-66.5), slightly, but not significantly, lower than that achieved using the murmur detection algorithm. Similarly, the network trained to predict moderate to severe MR achieved an AUC of only 57.5% (CI:52.3-62.6), which is slightly higher, but not significantly different from the murmur detection algorithms AUC of 55.8%. A significant issue was that the networks would sometimes fail to converge (i.e. fail to learn any features) and performance would never exceed that of a random guess. This would typically happen in 1-2 of the 8 cross validation runs. Thus, it is possible that the networks would achieve slightly higher values with additional tinkering with hyperparameters. However, the failures to converge and poor performance in the cases when they did converge indicates that there is probably a very weak signal-to-noise ratio for these targets.

Training on murmur grade vs directly on the aortic valve mean pressure gradient (6-fold cross validation on the entire dataset performed after analysis was completed) yielded AUC values (for mild or greater AS) of 97.2% and 95.1% respectively, a significant difference of 2.19% (CI: 4.2-0.20%; p=0.01).

## Comparison Between Continuous and Binary Labels

When training a murmur detection algorithm, we may model the problem as either a regression problem or as a classification problem. In the former approach, we model the annotated murmur grade as a continuous variable, and in the latter we model it as a binary variable, with prediction classes being "murmur" and "no murmur”. In the reduction of the data to binary form there is potentially a loss of information. For instance, if one recording is annotated as grade 1 and another as grade 0, it is appropriate for these two samples to assign different loss values to e.g. a prediction of grade 2. If we instead used binary labels and a cutoff for the positive class of e.g. grade 2, then the distinction between the labels would be lost, and a prediction of grade 2 (or a corresponding activation value) would receive the same loss in both cases. We suspect therefore that the regression approach might produce a better model, as it plausibly uses the training data more efficiently. Formally, our hypothesis is that the regression model will predict murmur grade≥2 more accurately than a classification model trained directly to predict such cases, with accuracy measured by the AUC.

We collect comparison data by performing 8-fold cross validation, using data from all positions jointly during training, with CV splits being based on participants ID to ensure independence between each training and validation set pair. For each fold, we compare AUC values, after which we use the paired t-test (two sided) to test for significant performance difference. We compare AUC for each position separately, as well as for all positions jointly (in each CV split, the 4 validation sets are joined into a single set for which the AUC is computed). In order to make the comparison as objective as possible, we use an automatic rule for training stoppage; training is stopped when the value of the loss function has failed to improve over 5 consecutive epochs (one check per epoch), with failure to improve being defined as failure to improve upon the preceding 4 values.

### Result

The regression model significantly outperformed the classification model. Testing the hypothesis that the regression network predicted murmur grade≥2 with a higher AUC than the classification network, the p-value obtained was 0.013 (**Table S3**). In this comparison, predictions for all recordings (positions 1-4) were used, with the success or failure of each prediction being treated as independent outcomes. Significant improvements were also seen for murmur prediction in positions 2, 3, and 4, but not in position 1 where the performance was not significantly different.

The aortic position had approximately twice as many cases of murmur-grade≥2 as any of the other positions. It is possible that, due to making less efficient use of the data, the classification network is more prone to overtraining than the regression network, and therefore it overfits to the aortic position, which would explain why we see significant outperformance in all other positions. In any case, it seems that modeling murmur grade as a continuous variable is likely a more efficient way of training a murmur detection algorithm than splitting the label data into two classes and modeling as a classification problem.

## Modified Version of Springers Segmentation Algorithm (patent pending)


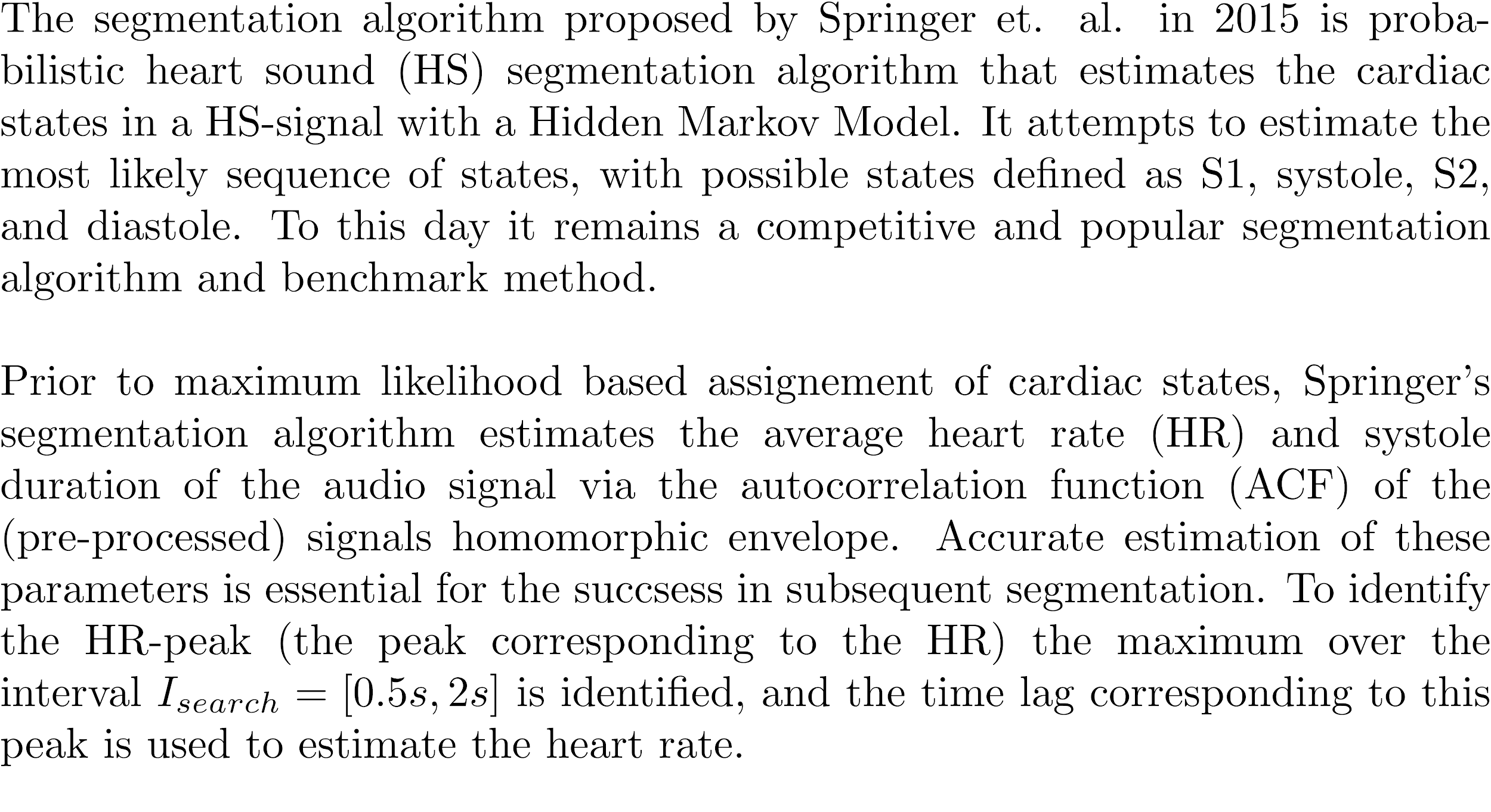


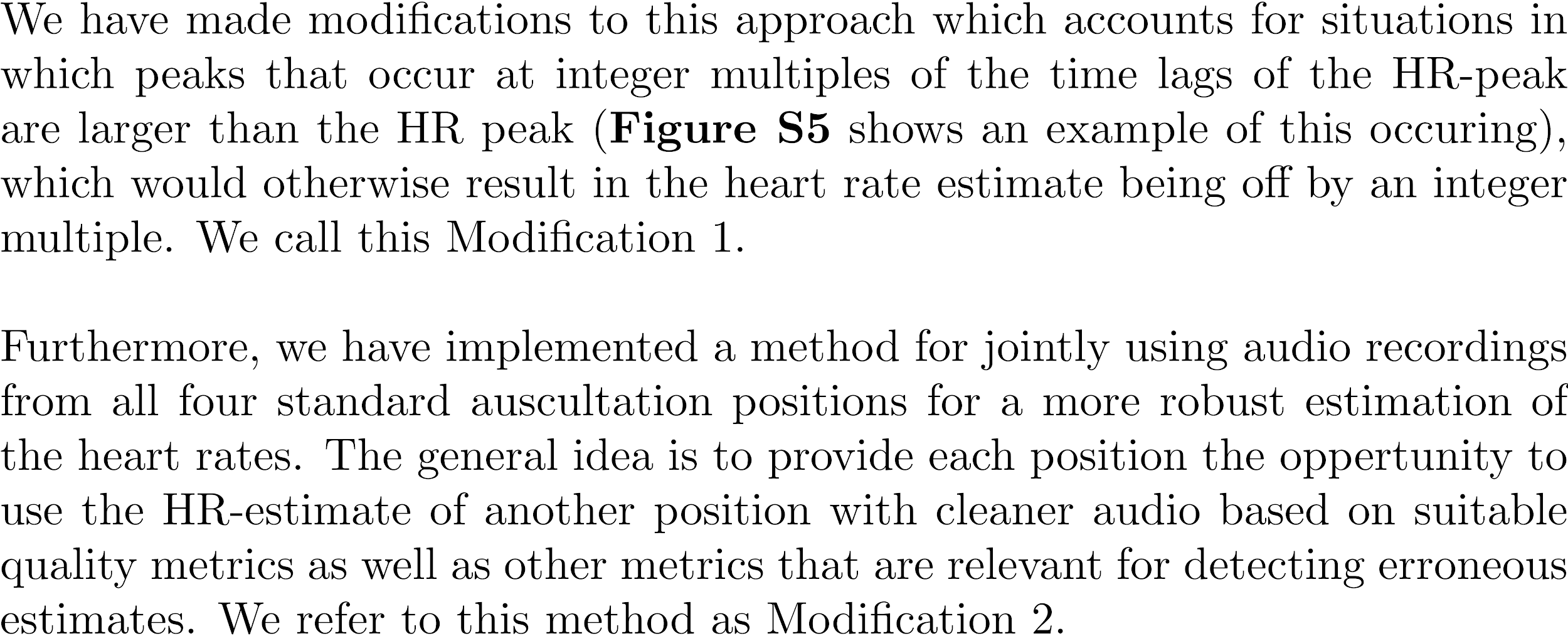


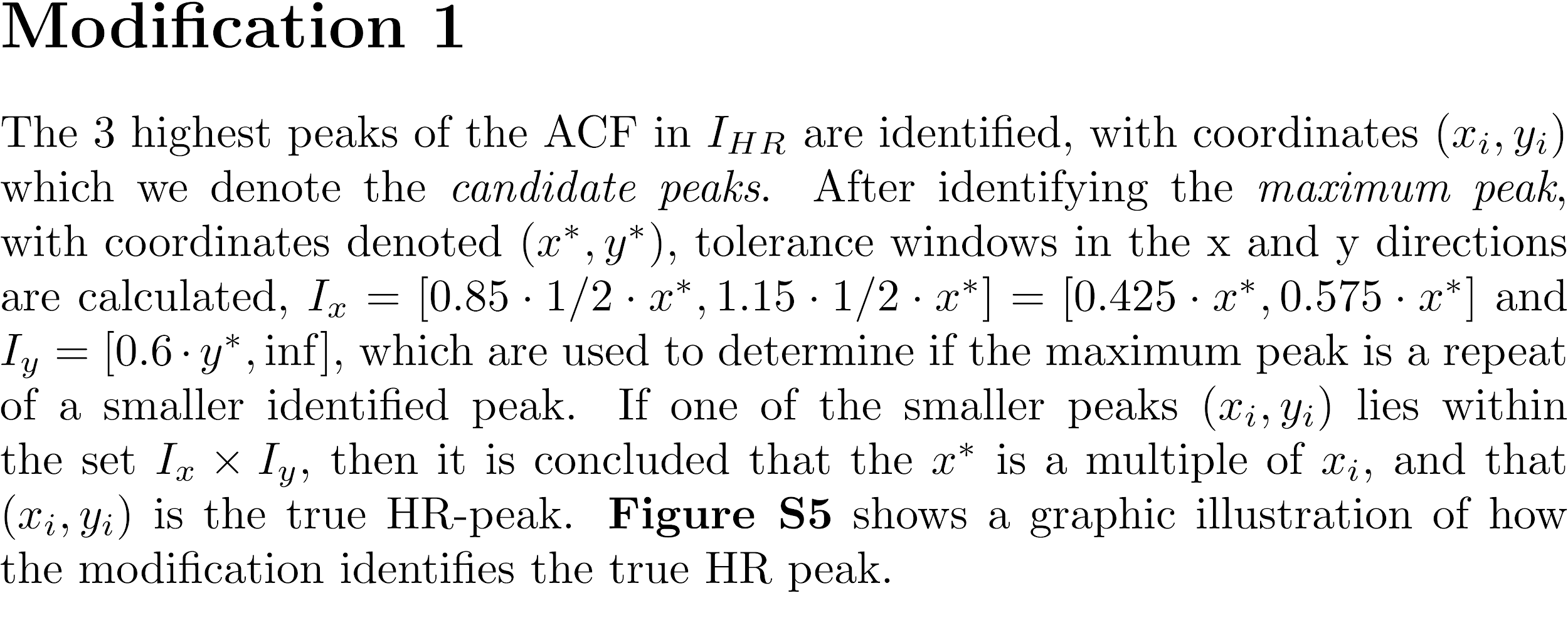


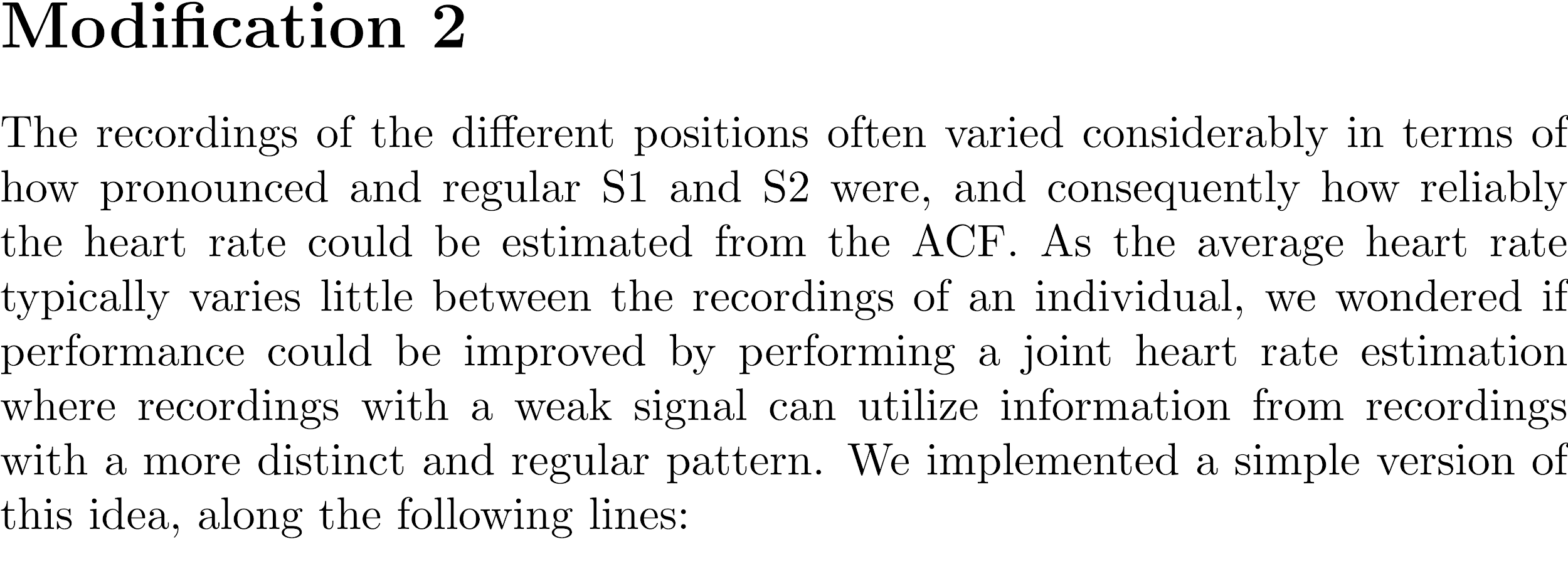


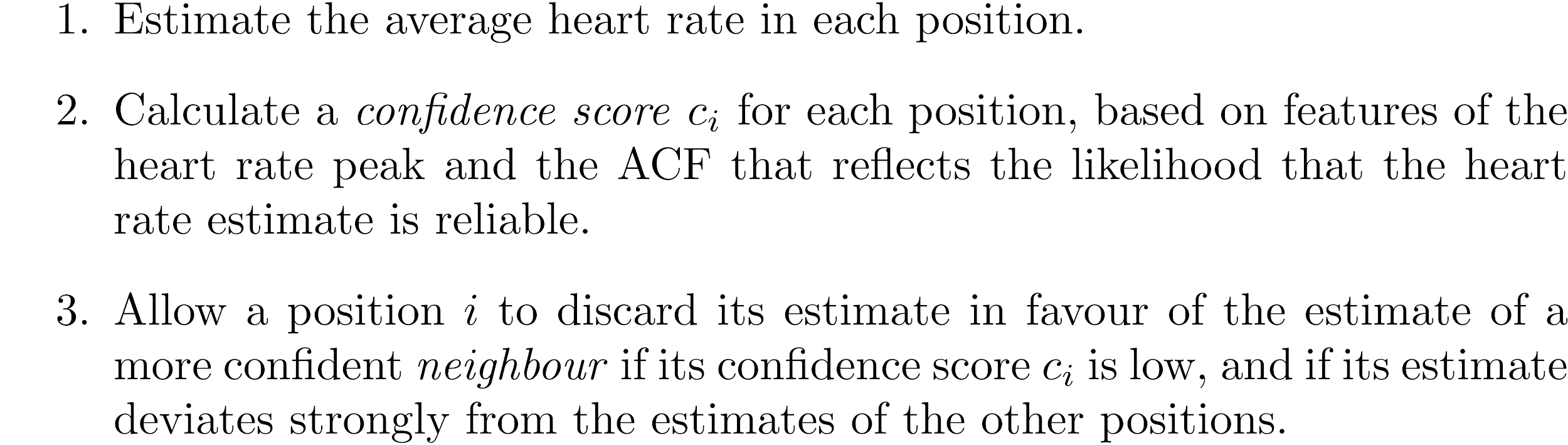


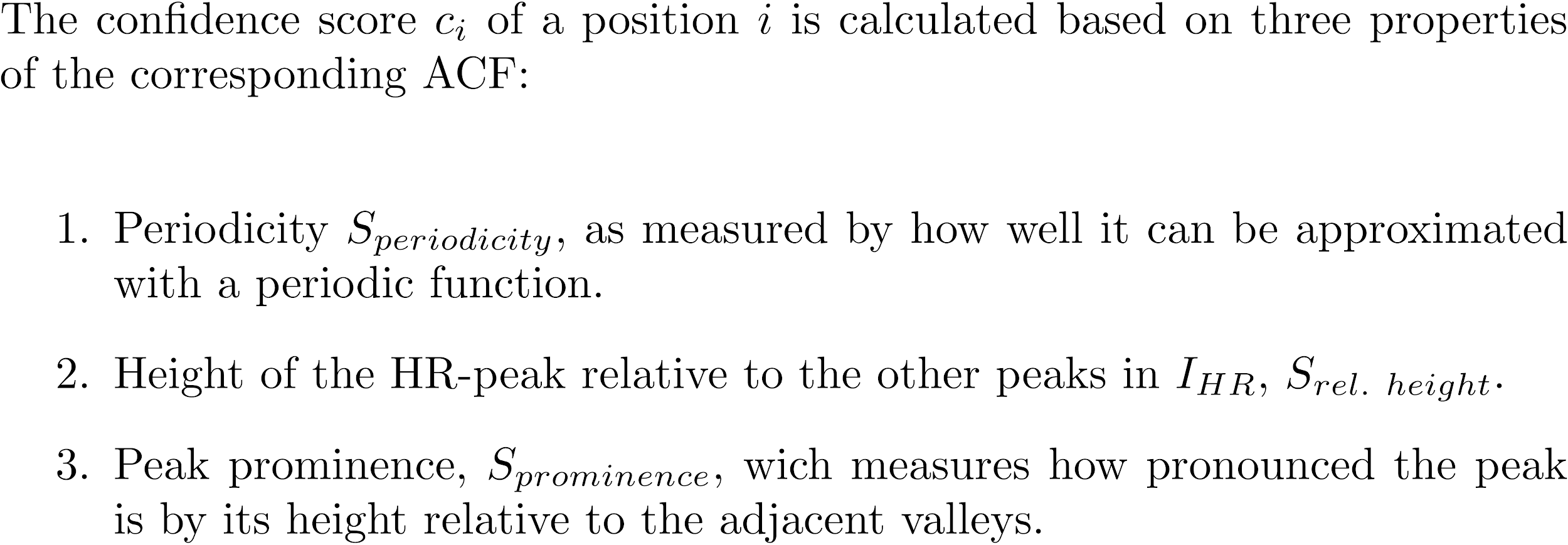


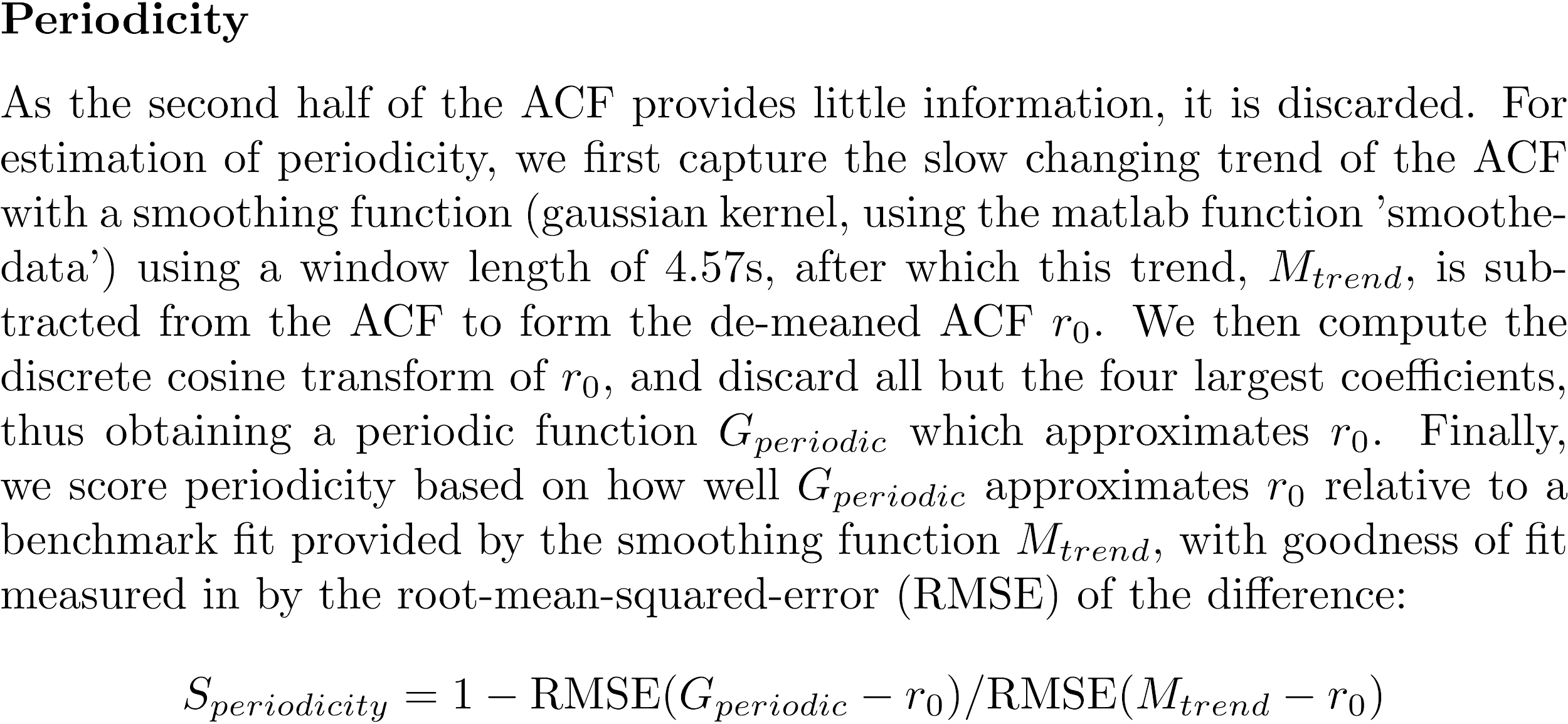


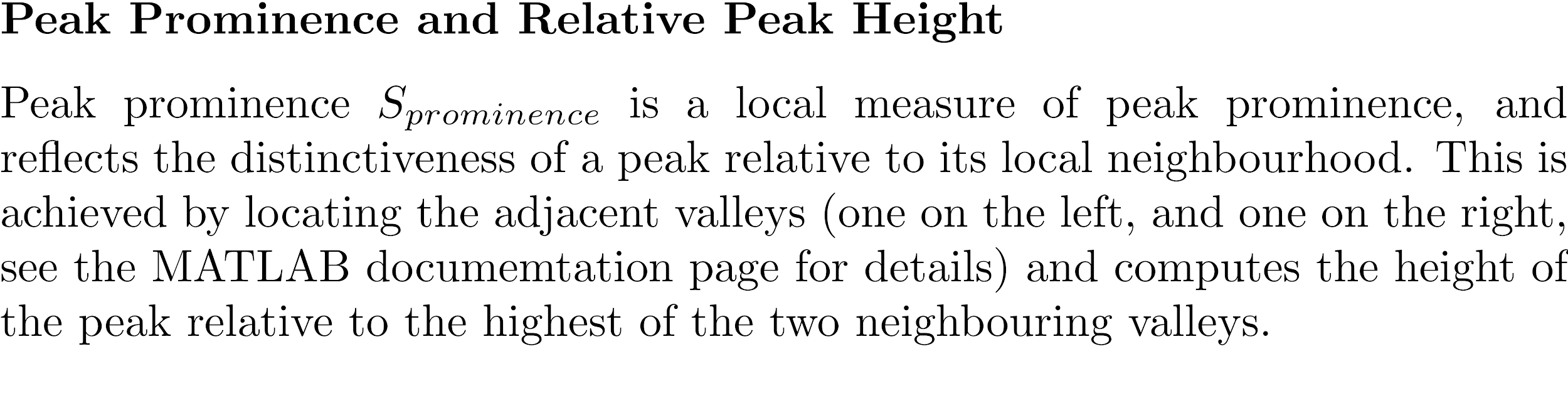


[
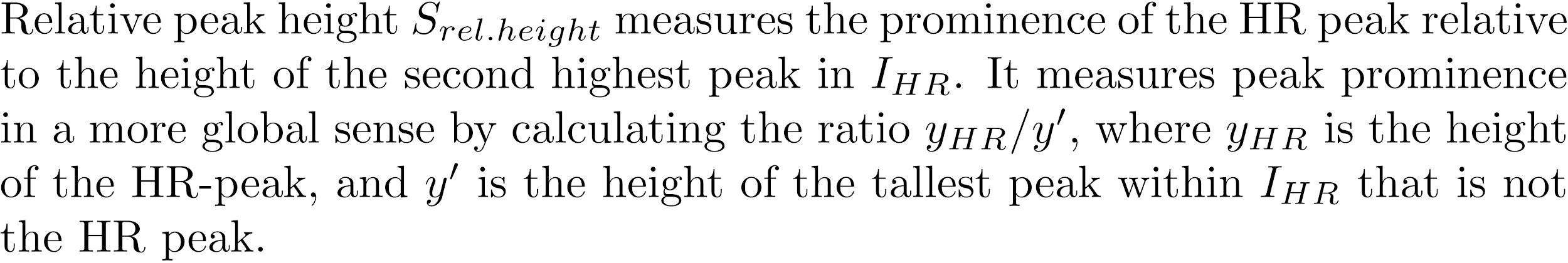
](#D2L_code_render_Relative_peak_height_$S_{rel._height}$_measures_the_prominence_of_the_HR_peak_relative_to_the_height_of_the_second_highest_peak_in_$I_{HR}$._It_measures_peak_prominence_in_a_more_global_sense_by_calculating_the_ratio_$y_{HR}/y'$,_where_$y_{HR}$_is_the_height_of_the_HR-peak,_and_$y'$_is_the_height_of_the_tallest_peak_within_$I_{HR}$_that_is_not_the_HR_peak.)


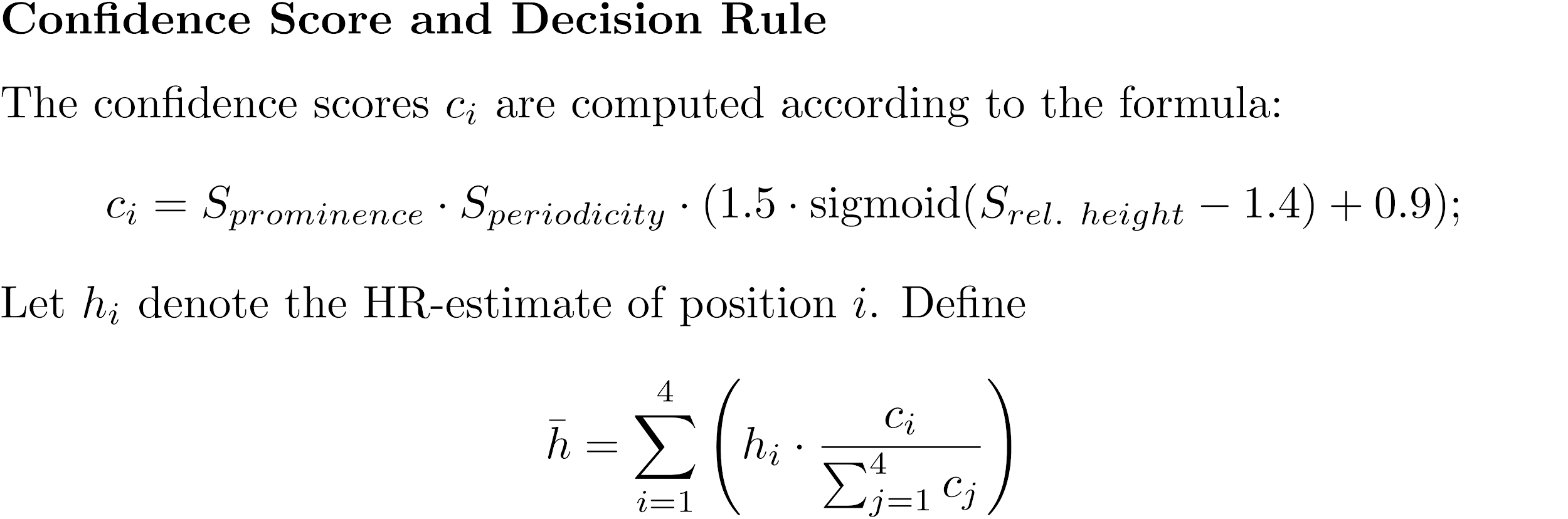


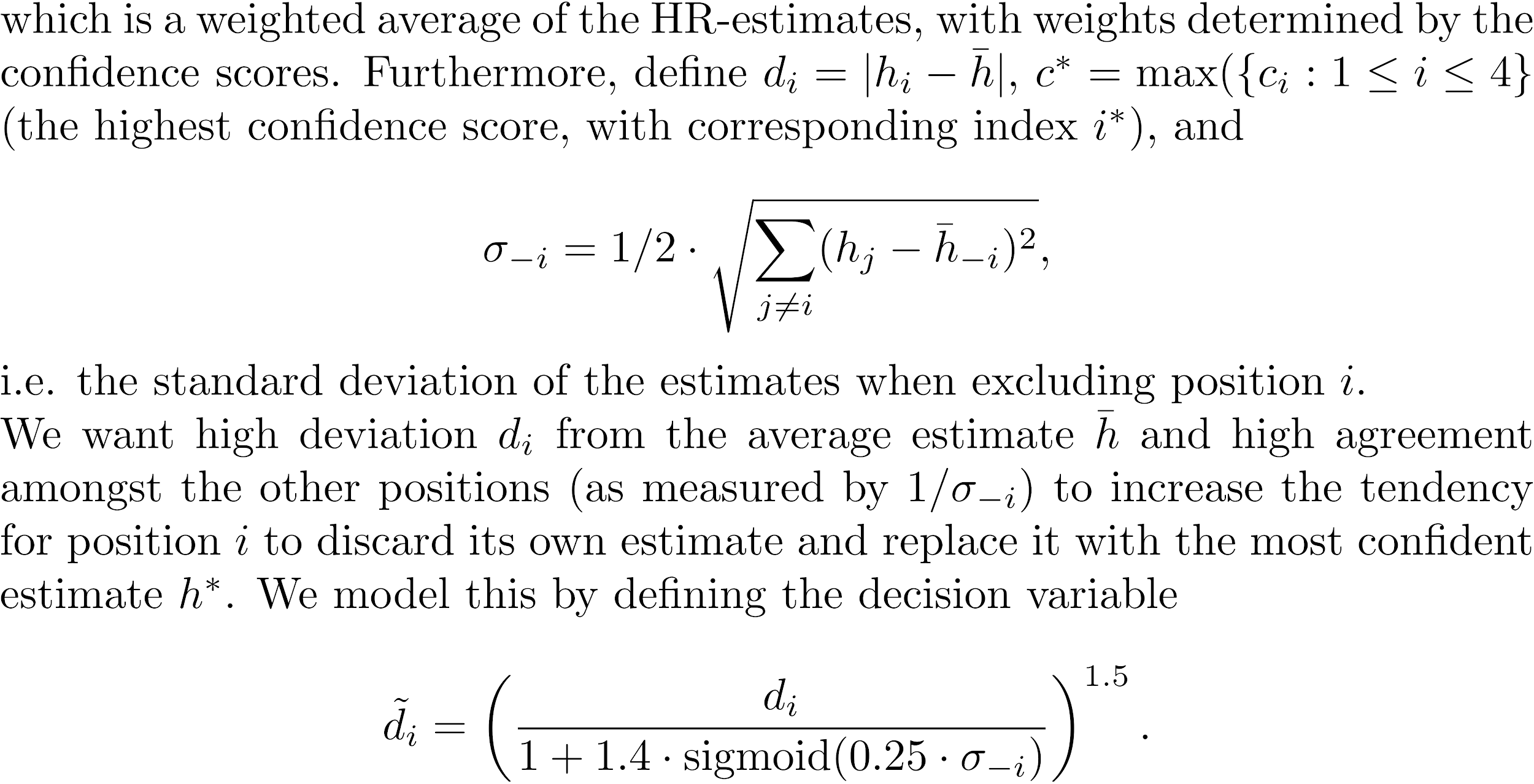


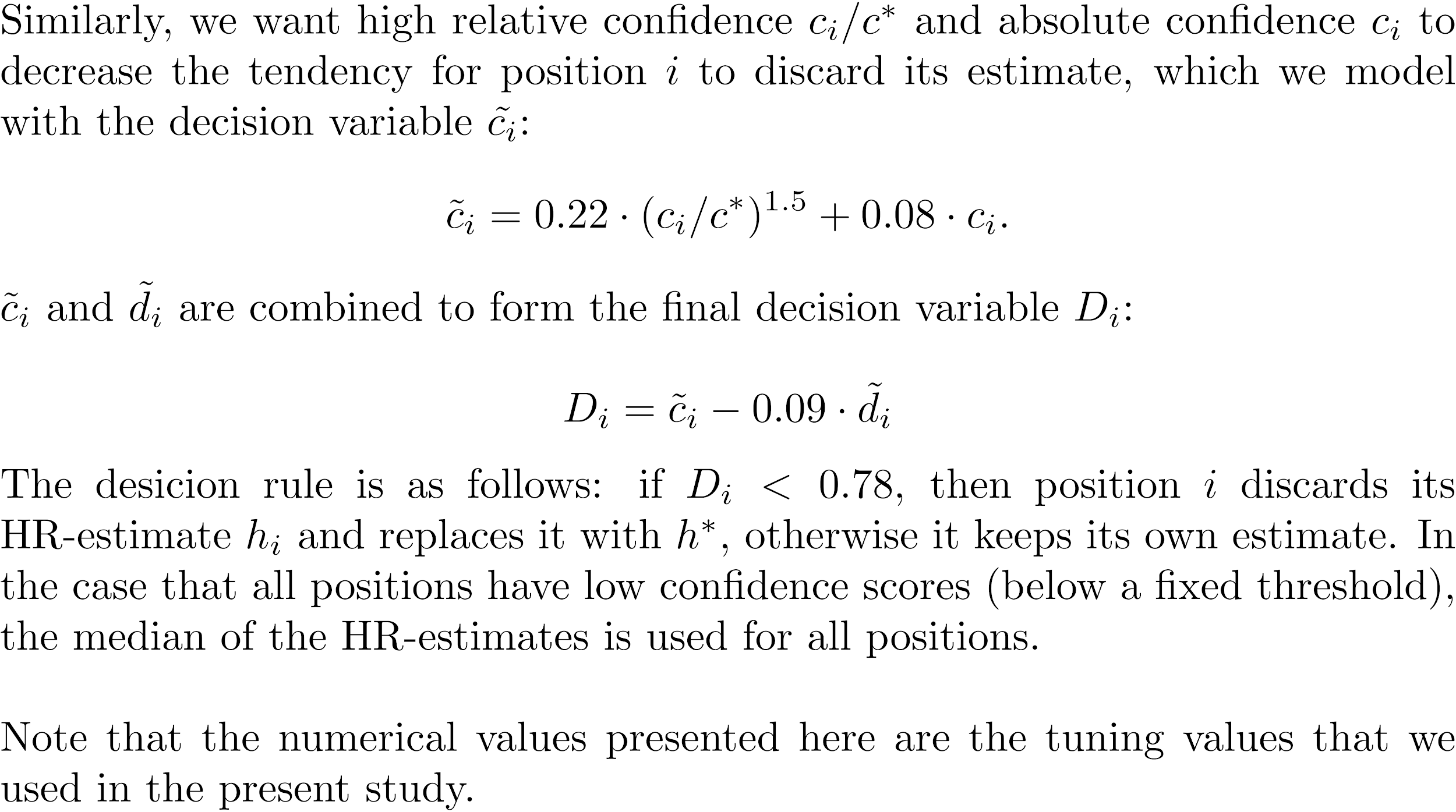

Supplement: Supplementary file 1 [file Datasheet1.docx]
